# KLHL3 deficiency in mice ameliorates obesity, insulin resistance, and nonalcoholic fatty liver disease by regulating energy expenditure

**DOI:** 10.1038/s12276-022-00833-w

**Published:** 2022-08-26

**Authors:** Ju-hong Jang, Jeong Woong Lee, Min Ji Cho, Byungtae Hwang, Min-Gi Kwon, Dong-Hwan Kim, Nam-Kyung Lee, Jangwook Lee, Young-Jun Park, Yong Ryoul Yang, Jinchul Kim, Yong-Hoon Kim, Tae Hyeon An, Kyoung-Jin Oh, Kwang-Hee Bae, Jong-Gil Park, Jeong-Ki Min

**Affiliations:** 1 Biotherapeutics Translational Research Center, Korea Research Institute of Bioscience & Biotechnology (KRIBB), Daejeon, Republic of Korea; 2 Department of Bioscience, KRIBB School of Bioscience, Korea University of Science and Technology (UST), Daejeon, Republic of Korea; 3 Environmental Disease Research Center, Korea Research Institute of Bioscience & Biotechnology (KRIBB), Daejeon, Republic of Korea; 4 Aging Research Center, Korea Research Institute of Bioscience & Biotechnology (KRIBB), Daejeon, Republic of Korea; 5 Laboratory Animal Resource Center, Korea Research Institute of Bioscience & Biotechnology (KRIBB), Daejeon, Republic of Korea; 6 Metabolic Regulation Research Center, Korea Research Institute of Bioscience & Biotechnology (KRIBB), Daejeon, Republic of Korea

**Keywords:** Obesity, Homeostasis

## Abstract

Obesity is a growing global epidemic that can cause serious adverse health consequences, including insulin resistance (IR) and nonalcoholic fatty liver disease (NAFLD). Obesity development can be attributed to energy imbalance and metabolic inflexibility. Here, we demonstrated that lack of Kelch-like protein 3 (KLHL3) mitigated the development of obesity, IR, and NAFLD by increasing energy expenditure. *KLHL3* mutations in humans cause Gordon’s hypertension syndrome; however, the role of KLHL3 in obesity was previously unknown. We examined differences in obesity-related parameters between control and *Klhl3*^*−/−*^ mice. A significant decrease in body weight concomitant with fat mass loss and improved IR and NAFLD were observed in *Klhl3*^*−/−*^ mice fed a high-fat (HF) diet and aged. KLHL3 deficiency inhibited obesity, IR, and NAFLD by increasing energy expenditure with augmentation of O_2_ consumption and CO_2_ production. Delivering dominant-negative (DN) *Klhl3* using adeno-associated virus into mice, thereby dominantly expressing DN-KLHL3 in the liver, ameliorated diet-induced obesity, IR, and NAFLD. Finally, adenoviral overexpression of DN-KLHL3, but not wild-type KLHL3, in hepatocytes revealed an energetic phenotype with an increase in the oxygen consumption rate. The present findings demonstrate a novel function of KLHL3 mutation in extrarenal tissues, such as the liver, and may provide a therapeutic target against obesity and obesity-related diseases.

## Obesity: A protein target for control

Mice that are genetically engineered to lack a protein involved in regulating energy expenditure are protected against the onset of obesity and the related problems of insulin resistance and non-alcoholic fatty liver disease. Jeong-Ki Min, Jong-Gil Park and colleagues at the Korea Research Institute of Bioscience & Biotechnology in South Korea, Daejon, discovered that the beneficial effect of the lack of the protein, called KLHL3, was due to an increase in energy expenditure. Mutations in the gene for KLHL3 are known to cause a variety of metabolic diseases in humans, including a form of high blood pressure called Gordon’s hypertension syndrome, but the protein’s role in human obesity has not been studied. The results suggest that drugs able to regulate the production or activity of KLHL3 might offer a new approach to treating obesity.

## Introduction

Obesity is delineated by substantial adipose tissue and ectopic fat distribution in the liver, muscles, and pericardial region. Obesity can evoke insulin resistance (IR) and nonalcoholic fatty liver disease (NAFLD) with local and systemic inflammatory responses, leading to increased morbidity and mortality^1–3^. IR, the failure of cells to respond normally to insulin signals to take up glucose, is a fundamental etiology of type 2 diabetes and is closely associated with the pathophysiology of NAFLD^4,5^. Obesity is driven by dysregulation of multiple factors, including physical activity, hormones, mitochondrial function, and metabolic flexibility^6–11^. Lifestyle changes such as reduced caloric intake and increased physical activity still make it difficult to resolve obesity in most patients, indicating an unmet need for medical intervention.

Kelch-like protein 3 (KLHL3), a member of the BTB-BACK-Kelch family, forms the E3 ubiquitin ligase complex with Cullin3 (CUL3)^12,13^. KLHL3 binds to with-no-lysine kinases (WNKs) and is involved in E3 ligase-mediated ubiquitination, which leads to WNK degradation^13,14^. In humans, *WNK1*, *WNK4*, *CUL3*, and/or *KLHL3* mutations manifest as Gordon’s syndrome, a heritable form of hypertension associated with salt-sensitive hypertension, hyperkalemia, metabolic acidosis, and thiazide sensitivity^15^. WNK4 (with-no-lysine kinase 4), a substrate of the CUL-KLHL3 E3 ligase complex, is an adipogenic factor whose deletion reduces high-fat (HF) diet-induced obesity in mice^16^. However, changes in body weight were similar between mice overexpressing WNK4 and wild-type (WT) mice^16^. In addition, Sasaki et al. reported that deficiency of KLHL3 did not have any effect on WNK levels in various tissues, with the exception of the kidney, suggesting that the CUL-KLHL3 E3 ligase complex is tissue-specific^17^. KLHL3 is ubiquitously expressed in normal human and mouse tissues^17,18^. However, evidence of the extrarenal functions of KLHL3, especially the regulation of obesity and obesity-related diseases, is limited.

In this study, we examined the role of KLHL3 in obesity, IR, and NAFLD using a mouse model. KLHL3 knockout (*Klhl3*^*−/−*^) mice showed reduced body weight gain through increasing energy expenditure leading to improvement in IR and NAFLD parameters in high-fat (HF) diet feeding conditions compared to that of control mice. The lack of KLHL3 also prevented the progression of NAFLD from NAFL to NASH in mice fed a methionine- and choline-deficient (MCD) diet. In addition to diet-induced obesity, KLHL3 deficiency in mice fed a normal chow (NC) diet gradually mitigated body weight gain from 4 to 10 months old. Delivering dominant-negative (DN) *Klhl3* to mice using adeno-associated virus (AAV), dominantly expressing DN-KLHL3 in the liver, ameliorated diet-induced obesity, IR, and NAFLD. Adenoviral overexpression of DN-KLHL3, but not WT-KLHL3, in hepatocytes revealed an energetic phenotype with an increase in the oxygen consumption rate (OCR). These findings suggest that KLHL3 modulation is a potential therapeutic target for the treatment of obesity and obesity-related diseases.

## Materials and methods

### Animals

This study conforms to the Guidelines on the Care and Use of Laboratory Animals (National Institutes of Health Publication no. 85-23, revised 1996). Animal study protocols were approved by the Institutional Animal Care and Use Committee of the Korea Research Institute of Bioscience and Biotechnology (KRIBB-AEC-21153). *Klhl3*^*−/−*^ mice were generated using the CRISPR/CAS9 system and were validated by PCR product sequencing after *Klhl3* gene amplification. The following primers were used for *Klhl3* genotyping: forward 5′-TTGGGTGAGAAGGTCGGGCT-3′ and reverse 5′-TAGTGTGACCCGGTGTCTGT-3′, which amplified an approximately 350 base pair (bp) DNA fragment.

To investigate the effects of KLHL3 on age-mediated obesity, 8-week-old male *Klhl3*^*+/+*^ and *Klhl3*^*−*/*−*^ mice were housed in a controlled environment for 30 weeks. Mice were given free access to NC and water for the duration of the experiment. For HF diet-induced obesity experiments, 8-week-old male and female *Klhl3*^*+/+*^ and *Klhl3*^*−*/*−*^ mice were placed on a HF diet (60% calories from fat; D12492, Research DIET Inc., New Brunswick, NJ, USA) for 12 weeks. To induce NASH, 8-week-old male *Klhl3*^+/+^ and *Klhl3*^*−*/*−*^ mice were placed on a MCD diet (TD.90262, Envigo, USA) for 6 weeks. All mice were housed in a controlled environment with a 12-h light/dark cycle in a pathogen-free facility. After the study, animals were anesthetized using isoflurane inhalation (3%) plus 1 L/min O_2_ and were euthanized by exsanguination.

### Body composition and indirect calorimetry

Mouse body composition was determined using dual-energy X-ray absorptiometry (DEXA, Lunar, GE Lunar Corp.). A DEXA scan was performed to identify body fat and bone area using a dedicated densitometer. Mice were placed in metabolic cages and allowed to acclimate for 24 h before energy expenditure, activity, and food intake were measured. Oxygen consumption (VO_2_), carbon dioxide production (VCO_2_), food consumption, and activity were automatically measured for 4 days using a computer-controlled indirect calorimetry system. Mice had ad libitum access to the respective diets and water during the study.

### Glucose and insulin tolerance tests

Mice were starved for 16 h or 6 h before the glucose tolerance test (GTT) or insulin tolerance test (ITT), respectively. Mice were given an intraperitoneal injection of 20% glucose (1 g/kg body weight, G8270; Sigma–Aldrich) or human insulin (0.75 units/kg body weight, I9728; Sigma–Aldrich). Blood samples were collected from the tail tips at the indicated time points for glucose measurement. Blood glucose levels were measured using an Accu-Check Active blood glucose meter (Roche).

### Metabolic parameters

Blood samples were collected via the retro-orbital sinus using heparinized capillary tubes (2501; KILBLE CHASE, USA). Plasma insulin was measured using an insulin ELISA kit (#90080; Crystal Chem) according to the manufacturer’s instructions. Plasma aspartate aminotransferase (AST) and alanine aminotransferase (ALT) were analyzed at the Laboratory Animal Resource Center (KRIBB, Daejeon, Korea). Lipids were extracted from liver tissues using a chloroform/methanol mixture (2:1 v/v), as described previously^19^. Hepatic triglyceride (TG) was measured using a Triglyceride Determination Kit (TR100; Sigma–Aldrich).

### Western blotting and immunoprecipitation (IP)

All animal tissue samples were washed in PBS and lysed with radioimmunoprecipitation assay buffer (50 mM Tris-HCl, 150 mM NaCl, 1 mM EDTA, 0.5% sodium deoxycholate, 1% Triton-X-100) or NP-40 buffer (50 mM Tris-HCl, 150 mM NaCl, 1% NP-40, pH 7.4) containing phosphatase inhibitor (50 mM beta-glycerophosphate, 50 mM NaF, 1 mM Na_3_VO_4_) and protease inhibitor cocktail (GenDEPOT, Houston, TX, USA). IP was performed with anti-IgG (Sc-3888, Santa Cruz Biotechnology) and anti-KLHL3 (16951-1-AP; Proteintech) in kidney and brain lysates. The immunoprecipitated sample proteins were quantified using a Pierce^TM^ BCA protein assay kit (23225; Thermo Fisher Scientific). The gastrocnemius muscle was collected from the hind legs, and its plasma membrane fraction was prepared using a series of differential centrifugations and a discontinuous sucrose gradient^20^. Protein lysates were subjected to western blotting with specific primary antibodies. After incubation with the primary antibodies, the membranes were incubated with species-specific peroxidase-conjugated secondary antibodies [1:10,000; cat. no. 31464 (rabbit) and 31430 (mouse), Thermo Fisher Scientific] at room temperature for 1 h. The membranes were detected with Amersham ECL Western Blotting Detection Reagent (RPN2106, GE) and visualized using a ChemiDoc imaging system (Bio-Rad). The primary antibodies are shown in Supplementary Table 1.

### Histology and immunohistochemistry

Tissue samples were fixed in 10% (v/v) phosphate-buffered formalin solution and incubated overnight. Paraffin-embedded tissue Section (4-μm thick) were used for hematoxylin and eosin (H&E) and Picro Sirius Red (ab150681; Abcam) staining, and frozen tissue sections (10-μm thick) were used for oil red O (O0625; Sigma) staining. After staining, photographs were captured under a light microscope (BX53F2; Olympus Corp, Tokyo, Japan). To calculate the white adipose tissue (WAT) lipid droplet size, H&E-stained WAT sections were photographed in 6–10 random locations at 20× or 40× magnification. The area of each adipocyte was measured using ImageJ software.

The severity of steatosis, lobular inflammation, and hepatocellular ballooning was scored using the NASH-Clinical Research Network criteria. Specifically, the amount of steatosis (percentage of hepatocytes containing fat droplets) was scored as 0 (<5%), 1 (5–33%), 2 (>33–66%), or 3 (>66%). Hepatocyte ballooning was classified as 0 (none), 1 (few), or 2 (many cells/prominent ballooning). Foci of lobular inflammation were scored as 0 (no foci), 1 (<2 foci per 200× field), 2 (2–4 foci per 200× field), or 3 (>4 foci per 200× field). The NAFLD activity score (NAS) was calculated from the grade of steatosis, inflammation, and ballooning. Fibrosis was scored as stage F0 (no fibrosis), F1a (mild, zone 3, perisinusoidal fibrosis), F1b (moderate, zone 3, perisinusoidal fibrosis), F1c (portal/periportal fibrosis), F2 (perisinusoidal and portal/periportal fibrosis), F3 (bridging fibrosis), or F4 (cirrhosis). For ease, F1a, F1b, and F1c fibrosis were all scored as 1.

For immunostaining, liver sections were incubated overnight with the following primary antibodies: rabbit anti-CD45 (ab10558; Abcam), rabbit anti-α-smooth muscle actin (α-SMA) (ab5694; Abcam), and rabbit anti-collagen type I (ab34710; Abcam). After incubation, antigens were visualized using Alexa 488 and 594 (Invitrogen, Carlsbad, CA) or biotinylated secondary antibodies with a 3,3′-diaminobenzidine substrate (Vector Laboratories, Burlingame, CA). DAPI or hematoxylin was used to label nuclei. Negative control tissues were prepared using IgG isotype control antibodies (Santa Cruz Biotechnology, Dallas, TX). Immunostaining images were captured using fluorescence and light microscopes (BX53F2; Olympus Corp).

### Quantitative real-time PCR (qRT–PCR) and reverse transcription (RT)-PCR

Total RNA was isolated from tissues using TRIzol reagent (15596026, Life Technologies). First-strand cDNA was synthesized using an M-MLV Reverse Transcriptase kit (N1705, C1101, N2515, U1518; Promega) following the manufacturer’s instructions. The cDNA was used as a template for RT–PCR. The quantitative RT–PCR analysis was performed using 2X Real-Time PCR smart mix (SRH72-M10h, SolGent, Republic of Korea) and EvaGreen (31000B500, SolGent) with primers. Target gene mRNA expression levels were normalized to *β-actin* or *18* *s* expression. The qRT–PCR and RT–PCR primer sequences are listed in Supplementary Table 2.

### Preadipocyte differentiation

Inguinal fat pads were obtained from 10-day-old C57BL/6 male mice to isolate primary preadipocytes. Inguinal fat pads were removed, minced, and digested using collagenase type I (1.5 mg/mL, Worthington Biochemical Corporation) at 37 °C for 45 min. Primary preadipocytes were cultured in Dulbecco’s modified Eagle’s medium (DMEM)/F12 GlutaMAX medium containing 15% FBS. Cells were placed in differentiation medium composed of DMEM containing 10% FBS and a differentiation cocktail (1 μM dexamethasone, 0.5 mM IBMX, and 10 μg/mL insulin) for 2 d (D1–2), transferred to DMEM with 10% FBS and 10 μg/mL insulin for 2 d (D3–4), and then maintained in DMEM with 10% FBS for an additional 4 d (D5–8). For the browning of adipocytes, differentiated adipocytes were treated with norepinephrine (1 μM) for 6 or 9 h.

### Proteomics

Proteins were extracted from the livers of NC- or MCD-fed mice (*n* = 3, pooled) using tissue lysis buffer [20 mM Tris-Cl (pH 8.0), 150 mM NaCl, 2 mM EDTA, 10% glycerol, 1% NP-40]. Protein lysates (100 μg) were digested using trypsin and analyzed using liquid chromatography (Easy-nLC II, Thermo Scientific) and mass spectrometry (MS) (LTQ Orbitrap Velos ETD, Thermo Scientific). Data were analyzed and compared with the UniProt Mouse Database using data search software (Sorcerer, Scaffold 4 Q + S).

### AAV and adenovirus

Recombinant AAV (AAV8.2 serotype vector) was used to express HA-KLHL3 R528H with enhanced green fluorescent protein (GFP) as a reporter under the control of a cytomegalovirus promoter. AAV vector production, purification, concentration, and titration were performed by Sirion (Martinsried, Germany). Mice were injected intravenously via the tail vein at a dose of 2.0 × 10^11^ vg/mouse and were fed a HF diet for 10 weeks.

Recombinant adenoviruses expressing flag-KLHL3 or HA-KLHL3 R528H with GFP were delivered using the pAdTRACK-CMV shuttle vector according to the manufacturer’s instructions. Recombinant adenoviruses were amplified in 293AD cells.

### Seahorse assay

Hep3B (3 × 10^3^ cells/well) or primary hepatocytes (1 × 10^4^ cells/well) isolated from *C57BL/6* or *Klhl3*^*−/−*^ mice were seeded into XFe24 microplates (Seahorse Bioscience, North Billerica, MA, USA). Transduction of adenovirus, including Ad-GFP, Ad-KLHL3, or Ad-DN-KLHL3 (KLHL3 R528H), was performed 24 h after plating the cells. The cells were subsequently incubated for 24 h at 37 °C with culture media. Three measurements were assessed under basal conditions and upon the addition of oligomycin (2.5 μM), FCCP (2 μM), and rotenone/antimycin A (0.5 μM) to measure the OCR (an indicator of mitochondrial respiration) and extracellular acidification (ECAR: an indicator of glycolysis) following the manufacturer’s instructions. All reagents were provided by the XF Cell Mito Stress Test Kit (Agilent Seahorse Biosciences). The results were normalized to the total protein OD values at the end of the experiments. After the seahorse assay, basal respiration, ATP production, nonmitochondrial respiration, proton leakage, maximal respiration, and energy maps were analyzed.

### Statistics

Data are expressed as the mean ± standard error of the mean. We performed an unpaired *t* test, one-way ANOVA, two-way ANOVA, and the Mann–Whitney U test to test statistical significance where appropriate. Statistical tests are described in the Figure Legends for each experiment. *P* values less than 0.05 were considered significant.

## Results

### KLHL3 deficiency mitigates HF diet-induced obesity, IR, and NAFLD by increasing energy expenditure

**Fig. 1:**
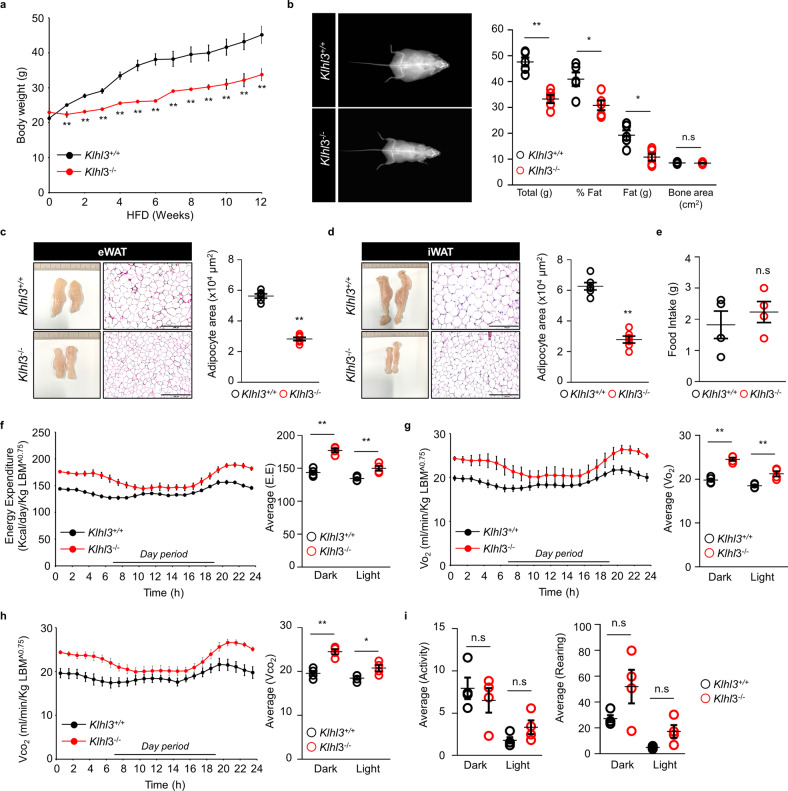
KLHL3 deficiency mitigates high-fat (HF) diet-induced obesity and decreases energy expenditure. **a** Body weight changes in *Klhl3*^*+/+*^ and *Klhl3*^*−/−*^ mice fed a HF diet for 12 weeks (*n* = 10). **b** Representative dual-energy X-ray absorptiometry imaging of mice (left) and quantification of body weight, body fat percentage, fat mass, and bone area (right) of *Klhl3*^*+/+*^ and *Klhl3*^*−/−*^ mice after 12 weeks on a HF diet (*n* = 5). **c**, **d** Representative macroscopic images and H&E-stained dissection images and adipocyte area in epididymal-white adipose tissue (eWAT) (c, *n* = 6) and inguinal-white adipose tissue (iWAT) (d, *n* = 6) of *Klhl3*^*+/+*^ and *Klhl3*^*−/−*^ mice fed a HF diet for 12 weeks. Scale bar, 500 μm. **e** Food intake of *Klhl3*^*+/+*^ and *Klhl3*^*−/−*^ mice during HF diet feeding (*n* = 4). **f**–**i** Energy expenditure (f, EE), VO_2_ **g**, VCO_2_ **h**, and physical activity levels **i** of *Klhl3*^*+/+*^ and *Klhl3*^*−/−*^ mice after 6 weeks of HF diet feeding (*n* = 4). EE, VO_2_, and VCO_2_ were normalized by lean body mass. Data are presented as the mean ± standard error of the mean *P < 0.05, **P < 0.01, n.s., not significant (Mann–Whitney U test for **a**–**e**, two-way ANOVA for **f**–**i**).

To investigate *Klhl3* gene functions in vivo, we generated two *Klhl3*^*−/−*^ mice carrying frameshift mutations, including a 2-bp insertion (2in) and a 17-bp deletion (17del). *Klhl3*^*−/−*^ mice were validated by DNA sequencing; immunoblot assays for KLHL3, NCC (sodium-chloride symporter), WNK1, and WNK4; and analysis of serum biochemistry parameters (Supplementary Fig. 1a–d and Supplementary Table 3). Next, we investigated whether the lack of KLHL3 in mice could modulate HF diet-induced obesity, IR, and NAFLD. Eight-week-old *Klhl3*^*+/+*^ and *Klhl3*^*−/−*^ (2in) male mice were placed on a HF diet for 12 weeks. HF diet-induced body weight gain was gradually suppressed in *Klhl3*^*−/−*^ mice compared to control mice; after 12 weeks, the difference in body weight between the two groups was more than 10 g (Fig. 1a). DEXA analysis showed that increased fat mass loss (19.27 vs. 10.80 g) was responsible for reducing HF diet-induced obesity in *Klhl3*^*−/−*^ mice (Fig. 1b). Additionally, *Klhl3*^*−/−*^ (2in) female mice and *Klhl3*^*−/−*^ (17del) male mice gained less body weight on the HF diet than did control mice (Supplementary Fig. 2a, b). DEXA analysis revealed that body weight and fat mass were reduced in *Klhl3*^*−/−*^ (2in) female mice compared to control mice (Supplementary Fig. 2c). After 12 weeks on a HF diet, the epidydimal-WAT (eWAT), inguinal-WAT (iWAT), brown adipose tissue (BAT) mass and adipocyte size were significantly lower in *Klhl3*^*−/−*^ (2in) male mice than in control mice (Fig. 1c, d and Supplementary Fig. 3a–c). However, the body weight and the ratios of eWAT, iWAT, and BAT mass to body weight were comparable between the 8-week-old *Klhl3*^*+/+*^ and *Klhl3*^*−/−*^ mice fed a normal chow diet (Supplementary Fig. 3d).

As a previous study reported that WNK4 is positively involved in adipocyte differentiation^16^, we investigated whether KLHL3 was involved in preadipocyte differentiation into adipocytes. Preadipocytes isolated from *Klhl3*^*+/+*^ and *Klhl3*^*−/−*^ mice were incubated in differentiation medium and evaluated by lipid staining and adipocyte marker analysis. Adipocyte lipid levels and adipocyte marker expression, including CCAAT/enhancer-binding protein alpha (C/EBPα), peroxisome proliferator-activated receptor-gamma (PPARγ), adipocyte protein (aP2), and adiponectin, were comparable between *Klhl3*^*+/+*^ and *Klhl3*^*−/−*^ differentiated adipocytes (Supplementary Fig. 4a–c). In addition, the mRNA levels of lipid metabolism- and thermogenesis-related genes revealed that KLHL3 deficiency in adipocytes did not contribute to a decrease in adipocyte size (Supplementary Fig. 4d).

**Fig. 2:**
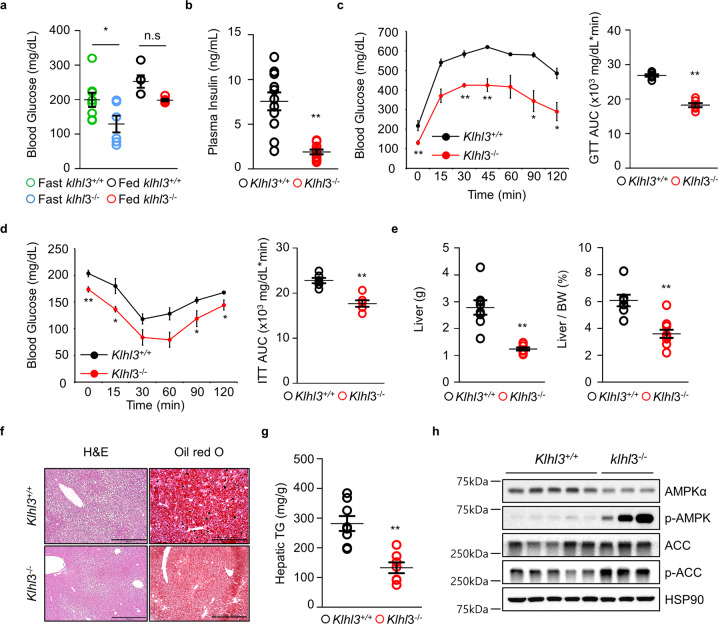
KLHL3 deficiency in mice fed a high-fat (HF) diet ameliorates insulin resistance and nonalcoholic fatty liver disease. **a**, **b** Levels of blood glucose (**a** *n* = 5–7) and plasma insulin (**b** *n* = 10–11) in *Klhl3*^*+/+*^ and *Klhl3*^*−/−*^ mice fed a HF diet for 12 weeks. **c** Glucose tolerance test (GTT) and the area under the curve (AUC) of *Klhl3*^*+/+*^ and *Klhl3*^*−/−*^ mice fed a HF diet for 8 weeks (*n* = 6). **d** Insulin tolerance test (ITT) and AUC of *Klhl3*^*+/+*^ and *Klhl3*^*−/−*^ mice fed a HF diet for 10 weeks (*n* = 6). **e** Liver weight and liver-to-body weight ratio in *Klhl3*^*+/+*^ and *Klhl3*^*−/−*^ mice fed a HF diet for 12 weeks (*n* = 7–10). **f** Representative images of liver sections stained with H&E (left) and oil red O (right); **g** hepatic triglyceride (TG) quantification of *Klhl3*^*+/+*^ and *Klhl3*^*−/−*^ mice fed a HF diet for 12 weeks (*n* = 7–8). Scale bar, 500 μm. **h** Immunoblot analysis of AMPKα, p-AMPK, ACC, and p-ACC in liver lysates of *Klhl3*^*+/+*^ and *Klhl3*^*−/−*^ mice fed a HF diet for 12 weeks. HSP90 served as a loading control. Data are presented as the mean ± standard error of the mean *P < 0.05, **P < 0.01, n.s., not significant (two-way ANOVA for **a**, Mann–Whitney U test for **b**–**e**, **g**).

Although daily food intake was comparable between the two groups, *Klhl3*^*−/−*^ mice fed a HF diet displayed an increase in lean body mass (LBM)-normalized energy expenditure, consumption of O_2_, and production of CO_2_ compared to those of control mice (Fig. 1e–h). *Klhl3*^*−/−*^ mice fed a HF diet showed no differences in locomotor activity and rearing responses compared to those of controls (Fig. 1i). Blood glucose levels were lower in *Klhl3*^*−/−*^ mice than in controls under fasting (129.5 vs. 199.6 mg/dL) and feeding (198.6 vs. 252.6 mg/dL) conditions (Fig. 2a). Plasma insulin levels (1.89 vs. 7.55 ng/mL) were markedly decreased in *Klhl3*^*−/−*^ mice compared to controls under feeding conditions (Fig. 2b). *Klhl3*^*−/−*^ mice also displayed improved glucose tolerance and insulin sensitivity, as validated by the GTT, ITT, and the levels of GLUT4 on the plasma membrane isolated from gastrocnemius muscles (Fig. 2c, d and Supplementary Fig. 5). Liver weights were significantly lower in *Klhl3*^*−/−*^ mice than in control mice, and HF diet-induced fatty livers (133 vs. 282 mg/g) were mitigated in *Klhl3*^*−/−*^ mice with activation of the AMPK signaling pathway (Fig. 2e–h).

### KLHL3 deficiency reduces MCD diet-induced NASH in mice

To test whether KLHL3 regulates NAFLD progression from NAFL to NASH, we fed a MCD diet to *Klhl3*^*+/+*^ and *Klhl3*^*−/−*^ mice for 6 weeks. MCD diet feeding induced changes in hepatic proteins involved in metabolic processes, immune system processes, and antioxidant activities (Supplementary Fig. 6). Proteomic analysis revealed that proteins encoded by the *Gck*, *Acadsb*, *Acsm5*, *Fahd2*, *Mavs*, *Irgm1*, and *Casp3* genes were commonly downregulated and that proteins encoded by the *H6pd*, *Gpi*, *Pgm3*, *Fads2*, *Acot4*, *Acot2*, *Gpi*, *Pgm3*, *S100a9*, *Gpx4*, and *Prdx4* genes were simultaneously upregulated in the livers of MCD-fed *Klhl3*^*+/+*^ and *Klhl3*^*−/−*^ mice compared with NC-fed *Klhl3*^*+/+*^ mice (Supplementary Fig. 6). However, differences in proteins encoded by the *Pdha2*, *Hdhd3*, *Sds*, *Nudt7*, *Decr2*, *Cbr4*, and *Prkaa1* genes involved in metabolic processes and *Rab10*, *Parp9*, *Lgals3*, *Fzd5*, *Stat1*, *Sbds*, *Tcea1*, *Oasl1*, *Mid2*, *Pir*, *Coro1a*, *Sqstm1*, *Nmi*, and *S100a8* genes involved in immune system processes and antioxidant activities were observed between MCD-fed *Klhl3*^*+/+*^ and *Klhl3*^*−/−*^ mice (Supplementary Fig. 6).

**Fig. 3:**
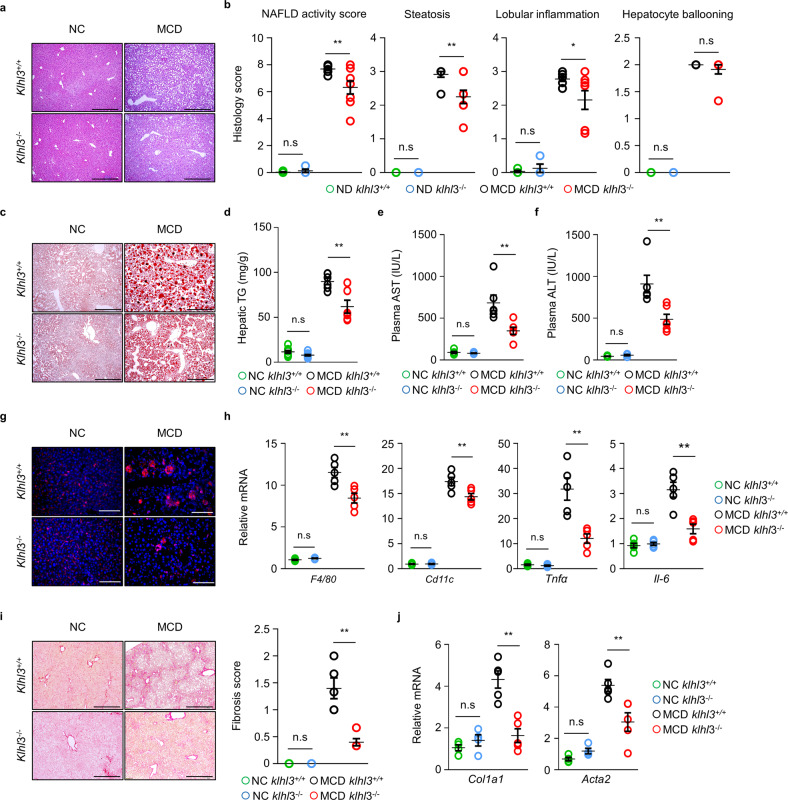
KLHL3 deficiency in mice prevents methionine- and choline-deficient (MCD) diet-induced nonalcoholic steatohepatitis. **a** Representative images and **b** quantification graphs of the NAFLD activity score, steatosis, lobular inflammation, and hepatocyte ballooning of liver sections stained with H&E from *Klhl3*^*+/+*^ and *Klhl3*^*−/−*^ mice fed a normal chow (NC) or MCD diet (*n* = 5–6). Scale bar, 200 μm. **c** Representative images of liver sections stained with oil red O from *Klhl3*^*+/+*^ and *Klhl3*^*−/−*^ mice fed a NC or MCD diet (*n* = 5–6). Scale bar, 200 μm. **d** Hepatic triglyceride (TG) quantification in *Klhl3*^*+/+*^ and *Klhl3*^*−/−*^ mice fed a NC or MCD diet (*n* = 6–9). **e** Plasma aspartate aminotransferase (AST) and **f** alanine aminotransferase (ALT) levels of *Klhl3*^*+/+*^ and *Klhl3*^*−/−*^ mice fed a NC or MCD diet (*n* = 4–6). **g** Representative images of CD45 (red) immunostained liver sections of *Klhl3*^*+/+*^ and *Klhl3*^*−/−*^ mice fed a NC or MCD diet (*n* = 5–6). Scale bar, 100 μm. **h** mRNA levels of the F4/80, Cd11c, Tnfα, and Il-6 genes in the livers of *Klhl3*^*+/+*^ and *Klhl3*^*−/−*^ mice fed a NC or MCD diet (*n* = 4–5). The qRT–PCR results were normalized to *18* *S*. **i** Representative images and the quantification graph of liver sections stained with Picrosirius red of *Klhl3*^*+/+*^ and *Klhl3*^*−/−*^ mice fed a NC or MCD diet (*n* = 3–6). Scale bar = 200 μm. **j** mRNA levels of *Col1a1* and *Acta2* in the livers of *Klhl3*^*+/+*^ and *Klhl3*^*−/−*^ mice fed a NC or MCD diet (*n* = 4–5). The qRT–PCR results were normalized to *18* *S*. Data are presented as the mean ± standard error of the mean *P < 0.05, **P < 0.01, n.s., not significant (two-way ANOVA for **b**, **d**–**f**, and **h**–**j**).

*Klhl3*^*+/+*^ and *Klhl3*^*−/−*^ mice fed the MCD diet for 6 weeks developed severe steatosis, lobular inflammation, and hepatocellular ballooning in the liver compared to controls fed a NC diet; however, the NAFLD activity was significantly decreased in the livers of *Klhl3*^*−/−*^ mice compared to the *Klhl3*^*+/+*^ mice when both groups were fed the MCD diet (Fig. 3a–d). Plasma AST and ALT levels were markedly increased in MCD-fed mice compared to NC-fed mice; however, the levels were significantly ameliorated in MCD-fed *Klhl3*^*−/−*^ mice compared with MCD-fed *Klhl3*^*+/+*^ mice (Fig. 3e, f). Consistently, MCD-fed mice exhibited increased immune cell accumulation in the liver compared to NC-fed mice, and colonies of CD45-positive cells in the liver were markedly decreased in MCD-fed *Klhl3*^*−/−*^ mice compared with MCD-fed *Klhl3*^*+/+*^ mice (Fig. 3g). MCD-fed *Klhl3*^*−/−*^ mice also had lower levels of mRNAs related to inflammation, including *F4/80*, *Cd11c*, *Tnfα*, and *Il-6* (Fig. 3h). Proteomic analysis revealed decreases in the levels of inflammatory response-regulating proteins encoded by the *Lgals3*, *Fzd5*, *Stat1*, *Oasl1*, *Mid2*, and *S100a8* genes in *Klhl3*^*−/−*^ mice fed the MCD diet compared to MCD-fed *Klhl3*^*+/+*^ mice (Supplementary Fig. 6).

Fibrosis and collagen deposition in the livers of MCD-fed mice were evaluated by Picrosirius red staining and immunohistochemistry for α-SMA and collagen type I. The MCD diet promoted fibrosis and collagen deposition in the livers of *Klhl3*^*+/+*^ mice; however, these effects were mitigated by KLHL3 deficiency (Fig. 3i and Supplementary Fig. 7a, b). These findings were corroborated by the analysis of gene expression, including *Col1a1* and *Acta2* (Fig. 3j).

### KLHL3 deficiency mitigated age-mediated obesity, IR, and NAFLD

**Fig. 4:**
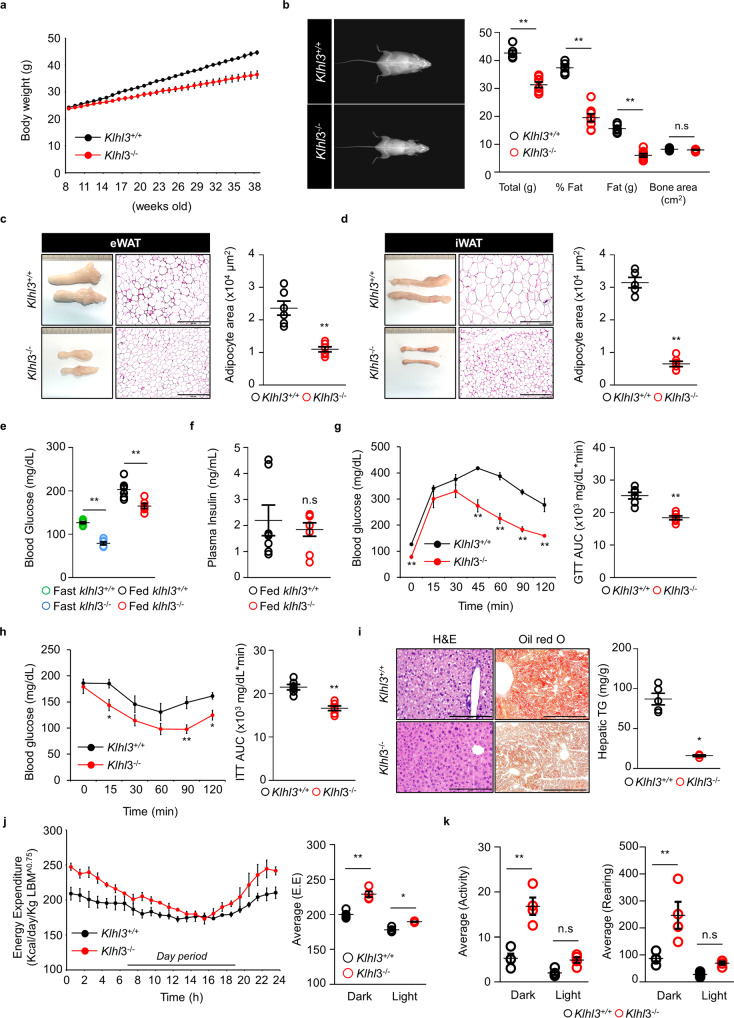
KLHL3 deficiency in mice prevents age-mediated obesity, insulin resistance, and nonalcoholic fatty liver disease. **a** Body weight changes in *Klhl3*^*+/+*^ and *Klhl3*^*−/−*^ mice fed a normal chow (NC) diet from 8 to 38 weeks old (*n* = 9–10). **b** Representative dual-energy X-ray absorptiometry imaging of mice (left) and quantification of body weight, body fat percentage, fat mass, and bone area (right) of *Klhl3*^*+/+*^ and *Klhl3*^*−/−*^ mice at 38 weeks old (*n* = 6–8). **c**, **d** Representative macroscopic and H&E-stained dissection images and adipocyte area in epididymal-white adipose tissue (eWAT) (**c** *n* = 6) and inguinal-white adipose tissue (iWAT) (**d** *n* = 5–6) of *Klhl3*^*+/+*^ and *Klhl3*^*−/−*^ mice fed a NC diet at 38 weeks old. Scale bar, 500 μm. **e** Levels of blood glucose (*n* = 6) and **f** plasma insulin (*n* = 7–8) in *Klhl3*^*+/+*^ and *Klhl3*^*−/−*^ mice fed a NC diet at 38 weeks old. **g** Glucose tolerance test (GTT) and the area under the curve (AUC) of 34-week-old *Klhl3*^*+/+*^ and *Klhl3*^*−/−*^ mice fed a NC diet (*n* = 6). **h** Insulin tolerance test (ITT) and AUC of 36-week-old *Klhl3*^*+/+*^ and *Klhl3*^*−/−*^ mice fed a NC diet (*n* = 6). **i** Representative images of liver sections stained with H&E (left) and oil red O (right); hepatic triglyceride (TG) quantification of *Klhl3*^*+/+*^ and *Klhl3*^*−/−*^ mice fed a NC diet at 38 weeks old (*n* = 4–5). Scale bar, 200 μm. **j** Energy expenditure (EE) and **k** physical activity levels of 32-week-old *Klhl3*^*+/+*^ and *Klhl3*^*−/−*^ mice fed a NC diet (*n* = 4). EE was normalized by lean body mass. Data are presented as the mean ± standard error of the mean. *P < 0.05, **P < 0.01, n.s., not significant (Mann–Whitney U test for **a**–**d** and **f**–**i**, two-way ANOVA for **e**, **j**, **k**).

Next, we investigated whether the lack of KLHL3 also ameliorates age-mediated obesity in mice fed a NC diet. At 8 weeks old, the body weights and food intake of *Klhl3*^*−/−*^ (2in) mice were comparable with those of control mice fed a NC diet; however, a discrepancy in body weight between the two groups appeared at 16 weeks (Fig. 4a and Supplementary Fig. 8a). At 10 months old, *Klhl3*^*−/−*^ mouse body weights were more than 15% lower than those of the control and were accompanied by fat mass loss. DEXA analysis confirmed that fat mass loss was the main contributor to the *Klhl3*^*−/−*^ body weight reduction (Fig. 4b). The weights of the eWAT, iWAT, BAT and liver were significantly reduced in aged *Klhl3*^*−/−*^ mice compared with control mice; however, reductions in tissue-to-body weight ratios were only observed in the eWAT and iWAT (Supplementary Fig. 8b–e). Consistently, the eWAT (1.09 vs. 2.36 × 10^4^ μm^2^) and iWAT (0.65 vs. 3.14 × 10^4^ μm^2^) adipocyte sizes were significantly lower in aged *Klhl3*^*−/−*^ mice than in control mice (Fig. 4c, d). Blood glucose levels were significantly lower in aged *Klhl3*^*−/−*^ mice than in control mice in fasting and fed conditions, but plasma insulin levels were similar between the two groups (Fig. 4e, f). The GTT revealed a notable improvement in glucose tolerance, and the ITT showed enhanced insulin sensitivity in aged *Klhl3*^*−/−*^ mice compared with control mice (Fig. 4g, h). In addition, the levels of GLUT4 in plasma membrane lysates isolated from skeletal muscle were higher in aged *Klhl3*^*−/−*^ mice than in controls (Supplementary Fig. 8f). Compared to control mice, aged *Klhl3*^*−/−*^ mice had less fat accumulation in the liver (87 vs. 16 mg/g), which was validated by H&E staining, oil red O staining, and biochemical analysis (Fig. 4i). Energy expenditure in aged *Klhl3*^*−/−*^ mice was significantly enhanced in both the light and dark phases (Fig. 4j). Locomotor activity and rearing responses were higher in aged *Klhl3*^*−/−*^ mice than in control mice during the dark phase but were comparable during the light phase (Fig. 4k).

### AAV-DN-*Klhl3* prevents HF diet-induced obesity, IR, and NAFLD

**Fig. 5:**
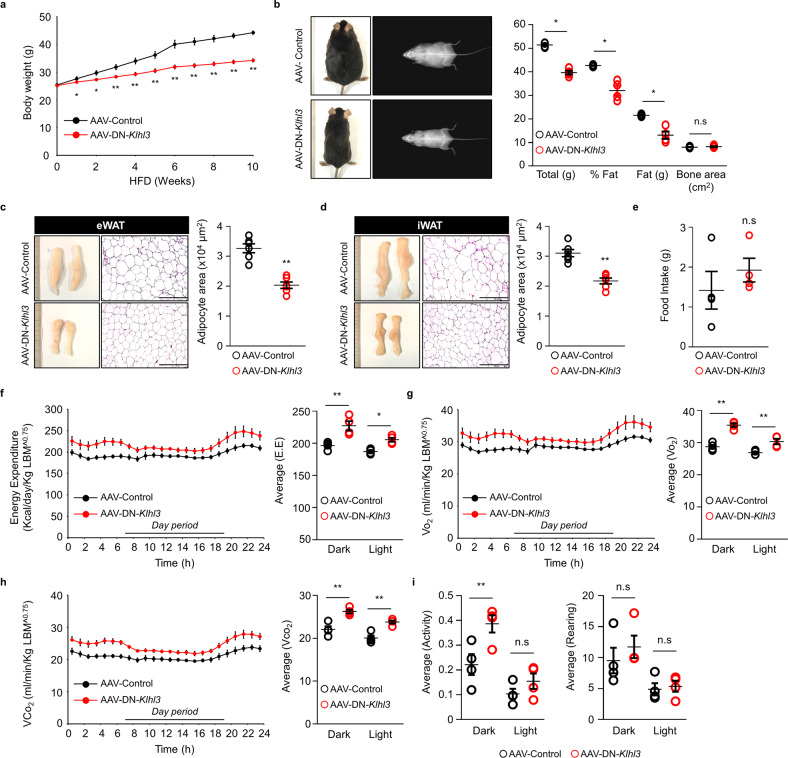
Adeno-associated virus (AAV) dominant-negative (DN)-*Klhl3* ameliorates high-fat (HF) diet-induced obesity by augmenting energy expenditure. **a** Body weight changes in AAV-Control- and AAV-DN-*Klhl3*-injected mice fed a HF diet for 10 weeks (*n* = 12). **b** Representative dual-energy X-ray absorptiometry imaging of mice (left) and quantification of body weight, body fat percentage, fat mass, and bone area (right) of AAV-Control- and AAV-DN-*Klhl3*-injected mice after 10 weeks on a HF diet (*n* = 4). **c**, **d** Representative macroscopic and H&E-stained dissection images and adipocyte area in epididymal-white adipose tissue (eWAT) (**c** *n* = 6) and inguinal-white adipose tissue (iWAT) (**d** *n* = 6) of AAV-Control- and AAV-DN-*Klhl3*-injected mice fed a HF diet for 10 weeks. Scale bar, 200 μm. **e** Food intake of AAV-Control- and AAV-DN-*Klhl3*-injected mice during HF diet feeding (*n* = 4). **f**–**i** Energy expenditure (**f** *EE*), VO_2_ **g**, VCO_2_ **h**, and physical activity **i** of AAV-Control- and AAV-DN-*Klhl3*-injected mice after 6 weeks of HF diet feeding (*n* = 4). EE, VO_2_, and VCO_2_ were normalized by lean body mass. Data are presented as the mean ± standard error of the mean *P < 0.05, **P < 0.01, n.s., not significant (Mann–Whitney U test for **a**–**e**, two-way ANOVA for **f**–**i**).

Point mutation of R528H in KLHL3 has been reported as a DN-KLHL3 that prevents KLHL3 interaction with substrates^21^, which was recapitulated in the immunoprecipitation assay (Supplementary Fig. 9a, b). We produced AAV-DN-*Klhl3* and AAV-Control to investigate the therapeutic efficacy of DN-KLHL3 in obesity and obesity-related diseases and injected the viruses into mice through the tail vein. After feeding a HF diet for 10 weeks, DN-KLHL3 expression in the liver was validated in AAV-Control- and AAV-DN-*Klhl3-*injected mice by immunoblot assays (Supplementary Fig. 9c). Interestingly, body weight discrepancies between AAV-Control- and AAV-DN-*Klhl3-*injected mice appeared within 3 weeks on the HF diet, resulting in a 9-gram difference after 10 weeks (Fig. 5a). Decreases in fat masses and eWAT (2.03 vs. 3.26 × 10^4^ μm^2^) and iWAT (2.1 vs. 3.1 × 10^4^ μm^2^) adipocyte sizes contributed to the reduced body weight in AAV-DN-*Klhl3-*injected mice compared to control mice (Fig. 5b–d and Supplementary Fig. 9d, e). Daily food intake was comparable between the two groups; however, energy expenditure, the consumption of O_2_, and the production of CO_2_, which were normalized by LBM, were significantly higher in AAV-DN-*Klhl3-*injected mice than in control mice (Fig. 5e–h). The locomotor activity and rearing response were comparable between the two groups, except locomotor activity in the dark phase, which was increased in AAV-DN-*Klhl3-*injected mice compared to control mice (Fig. 5i).

**Fig. 6:**
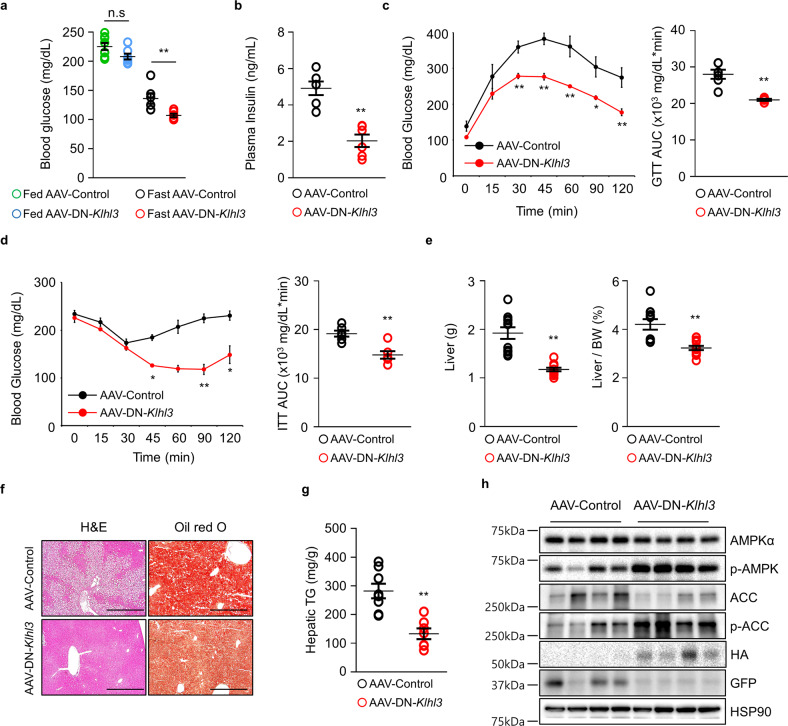
Adeno-associated virus (AAV) dominant-negative (DN)-*Klhl3* mitigates high-fat (HF) diet-induced insulin resistance and nonalcoholic fatty liver disease. **a** Levels of blood glucose (*n* = 5–6) and **b** plasma insulin (*n* = 10–11) in AAV-Control- and AAV-DN-*Klhl3*-injected mice fed a HF diet for 10 weeks. **c** Glucose tolerance test (GTT) and the area under the curve (AUC) of AAV-Control- and AAV-DN-*Klhl3*-injected mice fed a HF diet at 7 weeks (*n* = 6). **d** Insulin tolerance test (ITT) and AUC of AAV-Control- and AAV-DN-*Klhl3*-injected mice fed a HF diet at 9 weeks (*n* = 6). **e** Liver weight and liver-to-body weight ratio in AAV-Control- and AAV-DN-*Klhl3*-injected mice fed a HF diet for 10 weeks (*n* = 7–10). **f** Representative images of liver sections stained with H&E and oil red O; **g** hepatic triglyceride (TG) quantification of AAV-Control- and AAV-DN-*Klhl3*-injected mice fed a HF diet for 10 weeks (*n* = 7–8). Scale bar, 500 μm. **h** Immunoblot analysis of AMPKα, p-AMPK, ACC, p-ACC, HA, and GFP in liver lysates of AAV-Control- and AAV-DN-*Klhl3*-injected mice fed a HF diet for 10 weeks. HSP90 served as a loading control. Data are presented as the mean ± standard error of the mean *P < 0.05, **P < 0.01, n.s., not significant (two-way ANOVA for **a**, Mann–Whitney U test for **b**–**e**, and **g**).

Blood glucose levels were improved in AAV-DN-*Klhl3-*injected mice compared to controls in the fasted condition, and insulin plasma levels were markedly lower in AAV-DN-*Klhl3-*injected mice than in control mice after HF diet feeding (Fig. 6a, b). Glucose tolerance and insulin sensitivity were ameliorated in AAV-DN-*Klhl3-*injected mice compared with control mice after feeding a HF diet for 7 and 9 weeks, respectively (Fig. 6c, d). Moreover, liver weights and hepatic TG were significantly decreased with activation of the AMPK signaling pathway in AAV-DN-*Klhl3-*injected mice compared to control mice fed a HF diet (Fig. 6e–h).

### Overexpression of DN-KLHL3 in hepatocytes augmented mitochondrial function

**Fig. 7:**
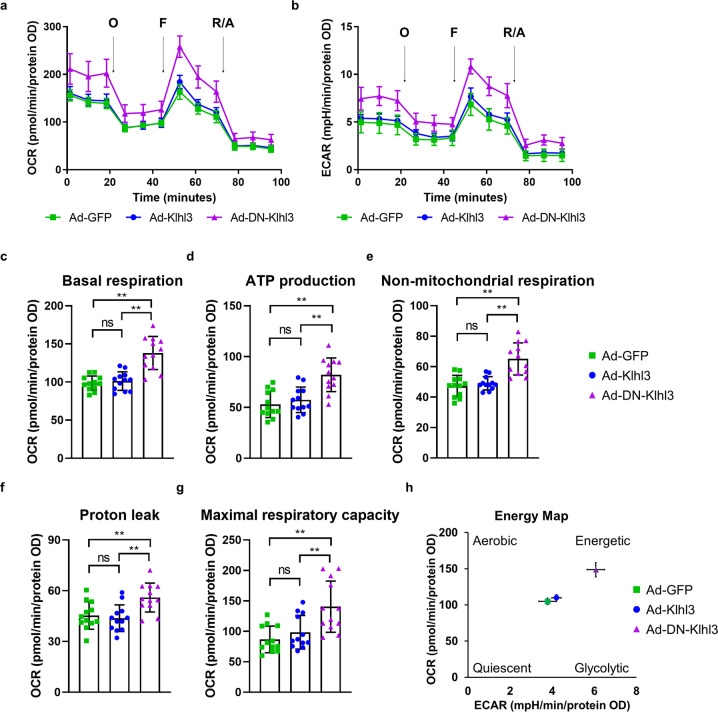
Adenoviral overexpression of dominant-negative (DN)-KLHL3 augmented mitochondrial function in primary hepatocytes isolated from *C57BL/6* mice. **a** The oxygen consumption rates (OCR) and **b** extracellular acidification ratio (ECAR) in primary hepatocytes (1 × 10^4^ cells/well) infected with Ad-GFP, Ad-KLHL3, or Ad-DN-KLHL3 were measured with a Seahorse XF analyzer. Cells were treated with oligomycin, FCCP, and rotenone + antimycin A at the indicated time points. **c** Statistical analysis of basal respiration in the OCR curve. **d** Statistical analysis of ATP production levels in the OCR curve. **e** Statistical analysis of nonmitochondrial respiration in the OCR curve. **f** Statistical analysis of the proton leak level in the OCR curve. **g** Statistical analysis of the maximal respiratory capacity in the OCR curve. **h** Energy map showing the increased energy status of primary hepatocytes overexpressing Ad-DN-KLHL3. Data represent three independent experiments. Data are presented as the mean ± standard error of the mean *P < 0.05, **P < 0.01, n.s., not significant (one-way ANOVA test for **c**–**g**).

DN-KLHL3 expression was predominantly expressed in the liver tissues of mice injected with AAV (Supplementary Fig. 9f). Therefore, the role of DN-KLHL3 in the energy metabolism of hepatocytes was further investigated using the Seahorse assay. Adenovirus-induced overexpression of DN-KLHL3, but not WT-KLHL3, enhanced the OCR and ECAR, in addition to increased basal respiration, respiration used for ATP production, nonmitochondrial respiration, proton leakage, and maximal respiratory capacity compared to those of controls in primary hepatocytes isolated from *C57BL/6* mice (Fig. 7a–g). Thus, overexpression of DN-KLHL3 in hepatocytes revealed the energetic phenotype (Fig. 7h). These results were recapitulated in human hepatoma Hep3B cells (Supplementary Fig. 10a–h). In addition, the expression levels of 115 proteins involved in mitochondrial function or organization were significantly changed in the livers of *Klhl3*^*−/−*^ mice fed a NC diet compared to those of controls, suggesting the KLHL3 deficiency-mediated regulation of mitochondrial function (Supplementary Fig. 11).

Finally, we verified whether the re-expression of KLHL3 in hepatocytes of *Klhl3*^*−/−*^ mice modulates energy metabolism. Adenovirus-mediated overexpression of WT-KLHL3 in primary hepatocytes isolated from *Klhl3*^*−/−*^ mice decreased the OCR and ECAR, followed by significant reductions in basal respiration, respiration used for ATP production, nonmitochondrial respiration, proton leakage, and maximal respiratory capacity compared to those of Ad-GFP controls (Supplementary Fig. 12a–h).

## Discussion

Obesity and obesity-related diseases represent substantial worldwide public health challenges^22–24^. Metabolic abnormalities in conjunction with obesity increase the risk of severe complications, including cardiovascular diseases, respiratory diseases, cancer, type 2 diabetes, and NAFLD. However, the prevalence of obesity continues to trend upward in most developed and developing countries^25–27^. Here, we demonstrated that KLHL3 deficiency in mice prevented diet- and age-induced obesity and mitigated IR and NAFLD. At 8 weeks old, the body weights of *Klhl3*^*−/−*^ mice fed a NC diet were comparable with those of control mice; however, KLHL3 deficiency contributed to a decrease in body weight with fat mass loss compared to that of controls in aged and HF diet-fed mice. Compared to controls, *Klhl3*^*−/−*^ mice exhibited increased energy expenditure, VO_2_, and VCO_2_. Diet- and age-induced obesity in control mice produced IR and NAFLD pathophysiology, but these parameters were notably alleviated in *Klhl3*^*−/−*^ mice. Moreover, KLHL3 deficiency prevented MCD diet-induced NAFLD progression from NAFL to NASH. Interestingly, we verified that DN-KLHL3 in mice fed a HF diet prevented obesity, IR, and NAFLD with increased energy expenditure, and overexpression of DN-KLHL3 in hepatocytes increased mitochondrial function with augmentation of the OCR. Our results provide evidence of the extrarenal role of KLHL3, which is linked to the development of obesity and obesity-related diseases induced by diet and/or aging.

Body weight loss and gain are modulated by an imbalance in food intake and energy expenditure^28^. As the *Klhl3* gene is abundantly expressed in the brain^17^, we verified whether KLHL3 deficiency affected food intake. Food intake was similar among *Klhl3*^*−/−*^ mice and control mice fed NC and HF diets, indicating that KLHL3 deficiency does not inhibit appetite in mice. However, the lack of KLHL3 in mice revealed increased energy expenditure in both HF diet- and age-induced obesity conditions. Although the physical activity level between *Klhl3*^*+/+*^ and *Klhl3*^*−/−*^ mice after feeding a HF diet was comparable, *Klhl3*^*−/−*^ mice at 10 months old exhibited increased physical activity compared to that of controls in the dark phase, suggesting that increased physical activity could partially contribute to enhanced energy expenditure in *Klhl3*^*−/−*^ mice. Thus, the function of KLHL3 in the brain must be elucidated to clarify the mechanism underlying increased physical activity in a future study. In addition to increased physical activity, an increase in mitochondrial function contributes to augmented energy expenditure. In the AAV-DN DN-*Klhl3* administration experiment, DN-KLHL3 was dominantly expressed in the liver and mitigated HF diet-induced obesity with increased energy expenditure, leading to the validation of DN-KLHL3 function in hepatocytes. Adenoviral overexpression of DN-KLHL3 in the primary hepatocytes of mice and human hepatoma cells enhanced the OCR and ECAR; however, WT-KLHL3 overexpression in hepatocytes showed similar levels of the OCR and ECAR compared to those of Ad-GFP controls, suggesting that DN-KLHL3 could specifically regulate mitochondrial function in hepatocytes. In addition, we confirmed that KLHL3 deficiency induced alterations in the expression profiles of proteins involved in mitochondrial function or organization in the livers of mice, indicating the regulation of mitochondrial function by KLHL3 modulation in autonomous hepatocytes. Among the altered proteins, a major functional protein related to the lack of KLHL3-mediated regulation of mitochondrial function in hepatocytes should be validated in a future study.

BAT and browning of adipose tissue are important for controlling whole-body energy expenditure^29^. KLHL3 deficiency in mice enhanced the expression of thermogenesis-related genes in BAT and iWAT but did not directly affect the browning of adipocytes (Supplementary Fig. 13a–c). These results suggested that the increase in energy expenditure of *Klhl3*^*−/−*^ mice during HF diet feeding may also result from the enhanced activation of BAT and browning of iWAT. Thus, the tissue-specific roles of KLHL3 in energy expenditure in mice could be validated using conditional knockout mice.

Here, we detected AMPK signaling pathway activation in the livers of *Klhl3*^*−/−*^ mice and AAV-DN-*Klhl3*-injected mice after feeding a HF diet. AMPK regulates cellular energy homeostasis by stimulating fatty acid oxidation, ketogenesis, and glucose uptake and inhibiting lipogenesis, cholesterol synthesis, and TG synthesis^30–33^. Therefore, we investigated whether KLHL3 interacts with the AMPK complex; however, we did not detect interactions between KLHL3 and AMPK isoforms, including AMPKα, AMPKβ1, and AMPKγ1 (data not shown). Additionally, KLHL3 or DN-KLHL3 overexpression in a hepatoma cell line did not affect the activity and half-life of AMPKα (data not shown). Therefore, we hypothesized that the activation of the AMPK signaling pathway in the liver might result from increased energy consumption in vivo.

KLHL3 deficiency or overexpression of mutant KLHL3 R528H prevented age- and diet-induced obesity and related diseases, suggesting that the function of KLHL3 as a substrate adapter contributes to accelerating obesity. KLHL3 promotes substrate ubiquitination through interaction with CUL3-based E3 ligases, and WNK kinases are well-known substrates for KLHL3;^13,14,17^ however, the role of KLHL3 interactions with other substrates in obesity is unknown. Although a previous study reported that WNK4 is positively involved in adipocyte differentiation^16^, the results of the present study showed that KLHL3 had no effects on the differentiation of primary preadipocytes, and Sasaki et al. reported that KLHL3-mediated degradation of WNKs was specific to the kidney^17^, ruling out the involvement of WNK kinases in the molecular mechanism.

Gordon syndrome (also called pseudohypoaldosteronism type II, PHAII) is a rare monogenic disease resulting from mutations to *WNK1*, *WNK4*, *CUL3*, and *KLHL3*. Takayhashi et al. reported that WNK4 is an adipogenic factor and that its deletion reduces diet-induced obesity in mice^16^; however, there have been no reports of a correlation between PHAII and the phenotype for obesity resistance in humans. Louis-Dit-Picard et al. reported the clinical characteristics of PHAII index cases with KLHL3 mutations; however, there were only BMI data for three individuals with homozygous *KLHL3* mutations, aged 2.5 and 3 months and 17 years^34^. Therefore, more cases with homozygous *KLHL3* mutations are needed to determine whether murine data can be translated to humans.

In conclusion, *Klhl3*^*−/−*^ mice displayed protective phenotypes that mitigated diet- and age-induced obesity, preventing IR and NAFLD progression. DN-*Klhl3* overexpression in mice fed a HF diet ameliorated body weight gain and obesity-related diseases. KLHL3 dysfunction caused by genetic mutations increased energy expenditure in mice, contributing to anti-obesity effects. These findings illustrate the novel role of KLHL3 in extrarenal function, providing a valuable therapeutic target against obesity and obesity-related diseases. However, KLHL3 mutation-mediated renal dysfunction and the lack of body mass index data of humans carrying homozygous *KLHL3* mutations remain to be addressed.

## Supplementary information

Supplementary data
