# Supplementary material for: KLHL3 deficiency in mice ameliorates obesity, insulin resistance, and nonalcoholic fatty liver disease by regulating energy expenditure
Source: Exp Mol Med. 2022 Aug 26;54(8):1250–61. doi: 10.1038/s12276-022-00833-w (PMC9440235; doi:10.1038/s12276-022-00833-w)

KLHL3 deficiency in mice ameliorates obesity, insulin resistance, and nonalcoholic fatty liver disease by regulating energy expenditure

Short title: KLHL3 deficiency ameliorates obesity with IR and NAFLD

Ju-hong Jang[1,6,#], Jeong Woong Lee[1,#], Min Ji Cho[1], Byungtae Hwang[1], Min-Gi Kwon[1,6], Dong-Hwan Kim[1], Nam-Kyung Lee[1], Jangwook Lee[1], Young-Jun Park[2], Yong Ryoul Yang[3], Jinchul Kim[3], Yong-Hoon Kim[4], Tae Hyeon An[5], Kyoung-Jin Oh[5], Kwang-Hee Bae[5], Jong-Gil Park[1,6,*], Jeong-Ki Min[1,6,*]

[1]Biotherapeutics Translational Research Center, [2]Environmental Disease Research Center, [3]Aging Research Center, [4]Laboratory Animal Resource Centre, [5]Metabolic Regulation Research Center, Korea Research Institute of Bioscience & Biotechnology (KRIBB), 125 Gwahak-ro, Yuseong-gu, Daejeon 34141, Republic of Korea.

[6]Department of Bioscience, KRIBB School of Bioscience, Korea University of Science and Technology (UST), 125 Gwahak-ro, Yuseong-gu, Daejeon 34141, Republic of Korea.

[#]These authors contributed equally to this work.

[*]Correspondence to Jeong-Ki Min, PhD., Biotherapeutics Translational Research Center, Korea Research Institute of Bioscience & Biotechnology, 125 Gwahak-ro, Yuseong-gu, Daejeon 34141, Republic of Korea; Tel: +82-42-860-4137; Fax: +82-42-860-4149; Email:

jekmin@kribb.re.kr

*Correspondence to Jong-Gil Park, PhD., Biotherapeutics Translational Research Center, Korea Research Institute of Bioscience & Biotechnology, 125 Gwahak-ro, Yuseong-gu, Daejeon 34141, Republic of Korea; Tel: +82-42-860-4122; Fax: +82-42-860-4149; Email: jonggilpark@kribb.re.kr

Supplementary Figure Legends

Supplementary Fig. 1 Generation of KLHL3 knockout mice using the CRISPR/Cas9 system.

a and **b** DNA sequences, frameshift mutation-induced first stop codon, and predicted premature amino acid sequences with a scheme in *Klhl3*$^{-/-}$ (a, 17del; b, 2in) mice. PCR product sequencing after amplification of *Klhl3* gene using genomic DNA from *Klhl3*$^{+/+}$ and *Klhl3*$^{-/-}$ (a, 17del; b, 2in) mice. The protospacer adjacent motif (PAM) sequence (CGG) is indicated in the black box on the genomic DNA sequence (top). **c** Immunoblot analysis for KLHL3 in immunoprecipitation (IP) samples. Antibodies for IgG and KLHL3 were used for IP in tissue lysates of *Klhl3*$^{+/+}$ and *Klhl3*$^{-/-}$ (2in, kidney and brain; 17del, brain) mice. **d** Immunoblot analysis for NCC, WNK4, and WNK1 in kidney lysates of *Klhl3*$^{+/+}$ and *Klhl3*$^{-/-}$ (2in and 17del) mice. HSP910 served as a loading control.

Supplementary Fig. 2 KLHL3 deficiency suppresses body weight gain in female and male mice fed a high-fat (HF) diet.

a Body weights changes in HF diet-fed *Klhl3*$^{+/+}$ and *Klhl3*$^{-/-}$ (2in) female mice for 12 weeks (n = 4–5). **b** Body weights changes in HF diet-fed *Klhl3*$^{+/+}$ and *Klhl3*$^{-/-}$ (17del) male mice for 12 weeks (n = 4). **c** Representative dual-energy X-ray absorptiometry imaging of mice (left) and quantification of body weight, body fat percentage, fat mass, and bone area (right) of *Klhl3*$^{+/+}$ and *Klhl3*$^{-/-}$ (2in) female after 12 weeks on an HF diet (n = 4–5). Data are presented

as mean ± standard error of the mean $^{*}P < 0.05$, $^{**}P < 0.01$, n.s., not significant (Mann–Whitney U test for a–c).

Supplementary Fig. 3 KLHL3 deficiency diet ameliorates obesity in mice fed a high-fat (HF) diet.

a–c Tissue weight (left) and tissue to body weight ratio (right) in epididymal (e)-white adipose tissue (WAT) (a), inguinal (i)-WAT (b), and brown adipose tissue (BAT) (c) of *Klhl3*$^{+/+}$ and *Klhl3*$^{-/-}$ (2in) male mice fed an HF diet for 12 weeks. **d** Body weight and the ratios of eWAT, iWAT, and BAT to body weight of *Klhl3*$^{+/+}$ and *Klhl3*$^{-/-}$ (2in) male mice fed a normal chow diet. Data are presented as mean ± standard error of the mean $^{*}P < 0.05$, $^{**}P < 0.01$, n.s., not significant (Mann–Whitney U test for a–d).

Supplementary Fig. 4 KLHL3 does not affect pre-adipocyte adipogenic differentiation.

a–d Primary pre-adipocytes were isolated from inguinal-white adipose tissue of *Klhl3*$^{+/+}$ and *Klhl3*$^{-/-}$ mice and cultured in the normal or differentiation medium. **a** Representative oil red O staining images of primary cells with and without adipogenic differentiation. **b** Immunoblot analysis of C/EBPα, PPARγ, and aP2 in lysates of undifferentiated and differentiated primary cells. β-actin served as a loading control. **c** *Adiponectin* mRNA expression was quantified by RT-PCR. *β-actin* mRNA expression served as a loading control. **d** mRNA levels of *Fas, Scd1, Acc1, Dgat2, Srebf1, Chrebp, Acox1, Cpt1a, Cpt2, Pparα, Ucp1, Prdm16, Pgc1a, CideA,* and *Dio2* genes in the pre-adipocytes and differentiated adipocytes isolated from *Klhl3*$^{+/+}$ and *Klhl3*$^{-/-}$ mice (n = 4–7). qRT-PCR results were normalized to *18S*. Data are presented as mean

± standard error of the mean *$P < 0.05$, **$P < 0.01$, n.s., not significant (two-way ANOVA test for d).

Supplementary Fig. 5 KLHL3 deficiency in mice fed a high fat (HF) diet ameliorates insulin resistance.

Immunoblots analysis for GLUT4 in lysates of plasma membrane isolated from gastrocnemius muscle of *Klhl3*$^{+/+}$ and *Klhl3*$^{-/-}$ mice after feeding an HF diet for 12 weeks. Na, K-ATPase served as a loading control.

Supplementary Fig. 6 Proteomic analysis of hepatic proteins isolated from *Klhl3*$^{+/+}$ and *Klhl3*$^{-/-}$ mice fed a normal chow (NC) or methionine- and choline-deficient (MCD) diet for 6 weeks.

Proteomic analysis of proteins involved in metabolic processes, immune system processes, and antioxidant activities. Differentially expressed proteins are shown as log fold-changes on the heat map.

Supplementary Fig. 7 KLHL3 deficiency in mice prevents MCD diet-induced liver fibrosis.

a and **b** Representative images of liver sections immunostained with antibodies against α-SMA (a, Scale bar = 100 μm) and collagen type I (b, Scale bar = 200 μm) of *Klhl3*$^{+/+}$ and *Klhl3*$^{-/-}$ mice fed an NC or MCD diet (n = 3–6).

Supplementary Fig. 8 KLHL3 deficiency in aged mice fed a normal chow (NC) diet ameliorates obesity.

a Food intake of *Klhl3*$^{+/+}$ and *Klhl3*$^{-/-}$ mice fed a normal chow (NC) diet. **b–e** Tissue weight (left) and tissue to body weight ratio (right) in epididymal (e)-white adipose tissue (WAT) (b), inguinal (i)-WAT (c), brown adipose tissue (BAT) (d), and liver (e) of *Klhl3*$^{+/+}$ and *Klhl3*$^{-/-}$ mice fed an NC diet at 38 weeks old. **f** Immunoblots analysis for GLUT4 in lysates of plasma membrane isolated from gastrocnemius muscle of *Klhl3*$^{+/+}$ and *Klhl3*$^{-/-}$ mice fed an NC diet at 38 weeks old. Na, K-ATPase served as a loading control. Data are presented as mean ± standard error of the mean $*P < 0.05$, $**P < 0.01$, n.s., not significant (Mann–Whitney U test for a–e).

Supplementary Fig. 9 Adeno-associated virus (AAV) dominant-negative (DN)-*Klhl3* suppresses high-fat (HF) diet-induced obesity in mice.

a Immunoblot analysis of WNK1 and KLHL3 in the whole cell lysates (WCL) and immunoprecipitation samples. Ad-Flag-KLHL3 (WT) and Ad-HA-KLHL3 (R528H) were infected in HEK293T cells, and KLHL3 proteins were precipitated by KLHL3 antibody. HSP90 served as a loading control. **b** Immunoblot analysis of WNK1, hemagglutinin (HA)-KLHL3, and flag-KLHL3. HEK293T cells were transfected by pcDNA-Flag-KLHL3 (WT) and/or pcDNA-HA-KLHL3 (R528H). Immunoprecipitation was performed using KLHL3 antibody. HSP90 served as a loading control. **c** Immunoblot analysis of HA-KLHL3 and green fluorescence protein (GFP) in the lysates of liver tissues of mice infected with AAV-Control

and AAV-DN-*Klhl3* after feeding an HF diet for 10 weeks. HSP90 served as a loading control. **d** and **e** Tissue weight (left) and tissue to body weight ratio (right) in epidydimal-white adipose tissue (eWAT) (d) and inguinal-white adipose tissue (iWAT) (e) of AAV-Control and AAV-DN-*Klhl3* injected mice fed an HF diet for 10 weeks. **f** Immunoblot analysis of HA-KLHL3 in the lysates of brain and liver tissues of mice infected with AAV-Control and AAV-DN-*Klhl3* after feeding an HF diet for 10 weeks. HSP90 served as a loading control. Data are presented as mean ± standard error of the mean *$P < 0.05$, **$P < 0.01$, n.s., not significant (Mann–Whitney U test for d, e).

Supplementary Fig. 10 Adenoviral overexpression of DN-KLHL3 augments the mitochondrial function in Hep3B cells.

a Oxygen consumption rates (OCR) and **b** extracellular acidification ratio (ECAR) of Hep3B cells (3×10^3 cells / well) infected with Ad-GFP, Ad-KLHL3, or Ad-DN-KLHL3 were measured with a Seahorse XF analyzer. Oligomycin, FCCP, and rotenone + antimycin A were treated at the indicated time points. **c** Statistical analysis of basal respiratory in the OCR curve. **d** Statistical analysis of ATP production levels in the OCR curve. **e** Statistical analysis of non-mitochondrial respiration in the OCR curve. **f** Statistical analysis of proton leak level in the OCR curve. **g** Statistical analysis of maximal respiratory capacity in the OCR curve. **h** Energy map showing an increased energetic status of Hep3B cells overexpressing Ad-DN-KLHL3. Data represent three independent experiments. Data are presented as mean ± standard error of the mean *$P < 0.05$, **$P < 0.01$, n.s., not significant (one-way ANOVA test for c–g).

Supplementary Fig. 11 KLHL3 deficiency in mice altered the expression profiles of hepatic proteins involved in mitochondrial function and/or organization.

Livers were isolated from *Klhl3*$^{+/+}$ and *Klhl3*$^{-/-}$ mice fed an NC diet. Proteomic analysis of proteins involved in mitochondrial function and/or organization. Differentially expressed proteins are shown as log fold-changes on the heat map.

Supplementary Fig. 12 Adenoviral overexpression of WT-KLHL3 decreases the mitochondrial function in primary hepatocytes of *Klhl3*$^{-/-}$ mice.

a Oxygen consumption rates (OCR) and **b** extracellular acidification ratio (ECAR) of *Klhl3*$^{-/-}$ primary hepatocytes (1×10^4 cells / well) infected with Ad-GFP or Ad-KLHL3 were measured with a Seahorse XF analyzer. Oligomycin, FCCP, and rotenone + antimycin A were treated at the indicated time points. **c** Statistical analysis of basal respiratory in the OCR curve. **d** Statistical analysis of ATP production levels in the OCR curve. **e** Statistical analysis of non-mitochondrial respiration in the OCR curve. **f** Statistical analysis of proton leak level in the OCR curve. **g** Statistical analysis of maximal respiratory capacity in the OCR curve. **h** Energy map showing a decreased energetic status of *Klhl3*$^{-/-}$ primary hepatocytes overexpressing Ad-KLHL3. Data represent three independent experiments. Data are presented as mean ± standard error of the mean *$P < 0.05$, **$P < 0.01$ (unpaired *t*-test for c–g).

Supplementary Fig. 13 Thermogenesis-related genes and the browning of adipocytes by the deficiency of KLHL3.

a mRNA levels of *Ucp1*, *Prdm16*, *Pgc1a*, *CideA*, and *Dio2* genes in BAT and iWAT of *Klhl3*$^{+/+}$

and *Klhl3*$^{-/-}$ mice fed an HF diet (n = 6–7). qRT-PCR results were normalized to *18S*. **b** Immunoblot analysis of UCP1 in BAT (top), and macroscopic and H&E stained sliced BAT images (bottom) of *Klhl3*$^{+/+}$ and *Klhl3*$^{-/-}$ mice fed an HF diet. Scale bar = 200 µm. **c** RT-PCR for *Ucp-1* in the differentiated adipocytes isolated from *Klhl3*$^{+/+}$ and *Klhl3*$^{-/-}$ mice with or without the stimulation of norepinephrine (NE). Actin levels served as a loading control. Data are presented as mean ± standard error of the mean **P* < 0.05, ***P* < 0.01, n.s., not significant (Mann–Whitney U test for a).

Supplementary Table 1. Antibodies

Antibody	SOURCE	Cat#
**β-actin**	AbClon	AbC2004
**HSP90**	Santa Cruz	sc-13119
**Flag**	Sigma-Aldrich	A8592
**HA**	Roche	11867423001
**AMPKα**	Cell Signaling Technology	#5832
**pAMPKα Thr172**	Cell Signaling Technology	#2535
**ACC**	Cell Signaling Technology	#3662S
**pACC Ser76**	Cell Signaling Technology	#3661
**KLHL3**	Proteintech	16951-1-AP
**NCC**	StressMarq	SPC402
**WNK4**	Millipore	07-2270
**mWNK1**	R&D systems	AF2849
**hWNK1**	Abcam	ab174854
**UCP1**	Abcam	ab10983
**GLUT4**	Abcam	ab654
**Na, K-ATPase**	Abcam	ab76020
**C/EBPα**	Cell Signaling Technology	#8178
**PPARγ**	Cell Signaling Technology	#2435
**aP2 (FABP4)**	Cell Signaling Technology	#2120
**GFP**	Abcam	ab290

Supplementary Table 2. qRT-PCR and RT-PCR primer sequences

Primer name	qRT-PCR Primer sequence	
Tnfα	Forward	CCC TCA CAC TCA GAT CAT CTT CT
	Reverse	GCT ACG ACG TGG GCT ACA G
Il6	Forward	TAG TCC TTC CTA CCC CAA TTT CC
	Reverse	TTG GTC CTT AGC CAC TCC TTC
F4/80	Forward	CTT TGG CTA TGG GCT TCC AGT C
	Reverse	GCA AGG AGG ACA GAG TTT ATC GTG
Cd11c	Forward	ACA CAG TGT GCT CCA GTA TGA
	Reverse	GCC CAG GGA TAT GTT CAC AGC
Cola1	Forward	AAG AAC AGC GTG GCC TAC ATG
	Reverse	GGG AGG TCT TGG TGG TTT TGT
Acta2	Forward	CAA TGG CTC TGG GCT CTG TAA
	Reverse	CTT TTG CTC TGT GCT TCG TCA
Ucp1	Forward	GTC GGT CCT TCC TTG GTG TA
	Reverse	GGG CCC TTG TAA ACA ACA AA
Prdm16	Forward	CAG CAC GGT GAA GCC ATT C
	Reverse	GCG TGC ATC CGC TTG TG
Pgc1a	Forward	ATG TGT CGC CTT CTT GCT CT
	Reverse	ATC TAC TGC CTG GGG ACC T
CideA	Forward	TGC TCT TCT GTA TCG CCC AGT
	Reverse	GCC GTG TTA AGG AAT CTG CTG
Dio2	Forward	CAG TGT GGT GCA CGT CTC CAA TC
	Reverse	TGA ACC AAA GTT GAC CAC CAG
Fas	Forward	CCC TTG ATG AAG AGG GAT CA
	Reverse	GAA CAA GGC GTT AGG GTT GA
Scd1	Forward	TTC TTG CGA TAC ACT CTG GTG C
	Reverse	CGG GAT TGA ATG TTC TTG TCG T
Acc1	Forward	CTC CAG GAC AGC ACA GAT CA

	Reverse	TGA CTG CCG AAA CAT CTC TG
Dgat2	Forward	GAA GCT GCC CGC AGC GAA AA
	Reverse	TCT TGG GCG TGT TCC AGT CAA
Srebf1	Forward	CAG CTC AGA GCC GTG GTG A
	Reverse	TTG ATA GAA GAC CGG TAG CGC
Chrebp	Forward	CCA GCC TCA AGG TGA GCA AA
	Reverse	CAT GTC CCG CAT CTG GTC A
Acox1	Forward	TCA ACA GCC CAA CTG TGA CT
	Reverse	GGC CGA TAT CCC CAA CAG TG
Cpt1a	Forward	CTC CGC CTG AGC CAT GAA G
	Reverse	CAC CAG TGA TGA TGC CAT TCT
Cpt2	Forward	CAG CAC AGC ATC GTA CCC A
	Reverse	TCC CAA TGC CGT TCT CAA AAT
Ppara	Forward	CTG CAG AGC AAC CAT CCA GA
	Reverse	TGA TGA CCT GTA CGA GCT GC

Primer name	RT-PCR Primer sequence	
Adiponectin	Forward	AGG AAA GGA GAG CCT GGA G
	Reverse	CCA GAC TTG GTC TCC CAC CT
Ucp-1	Forward	CCC ACT AGC AGC TCT TTG GA
	Reverse	CTG TGG AGC AGC TCA AAG GT
Actin	Forward	AGC CAT GTA CGT AGC CAT CC
	Reverse	CTC TCA GCT GTG GTG GTG AA

Supplementary Table 3. Serum biochemistry parameters

**Serum biochemistry parameter**	**Mean ± SEM**		***p*-value**
	***Klhl3*$^{+/+}$ mice (n = 7)**	***Klhl3*$^{-/-}$ mice (n = 7)**	
Na^+ (mmol/L)	145.31 ± 0.65	146.93 ± 1.17	ns
K^+ (mmol/L)	7.33 ± 0.09	8.21 ± 0.24	**
Cl^- (mmol/L)	115.28 ± 0.74	118.72 ± 1.02	*P*=0.053
Glucose (mg/dl)	186.69 ± 4.99	171.48 ± 11.62	ns
Blood urea nitrogen (mg/dl)	19.52 ± 1.44	16.51 ± 0.73	ns
Creatinine (mg/dl)	0.11 ± 0.01	0.06 ± 0.01	**

*P < 0.05 and **P < 0.01 (Mann–Whitney U test)

Supplementary Fig. 1

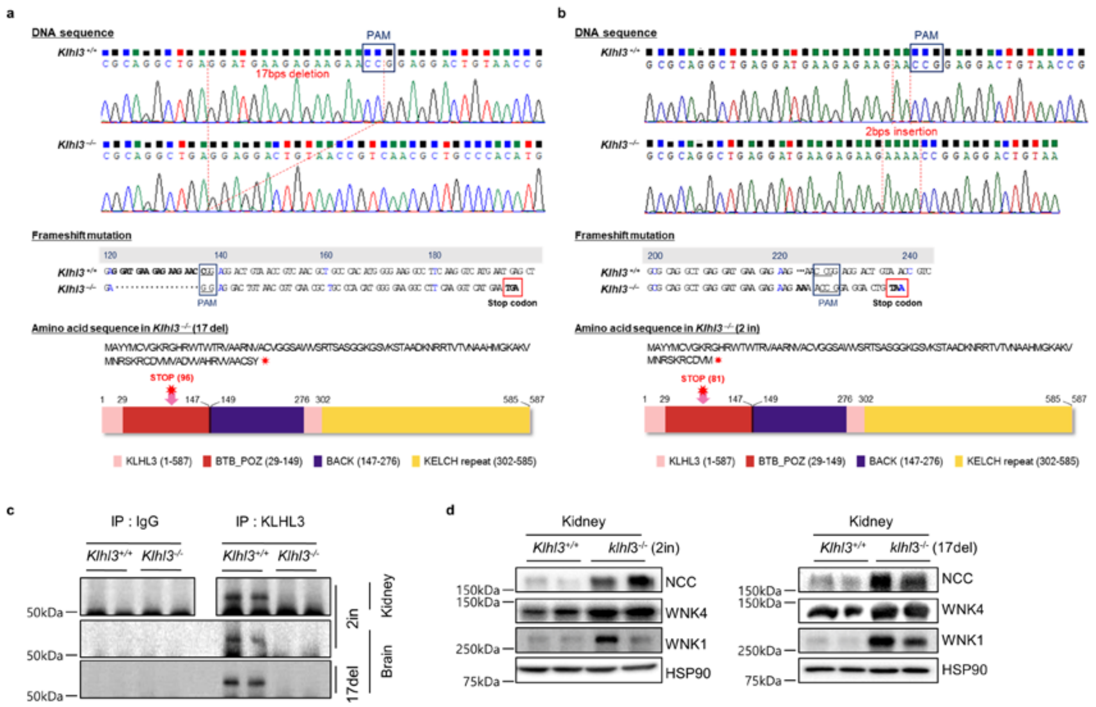

Supplementary Fig. 2

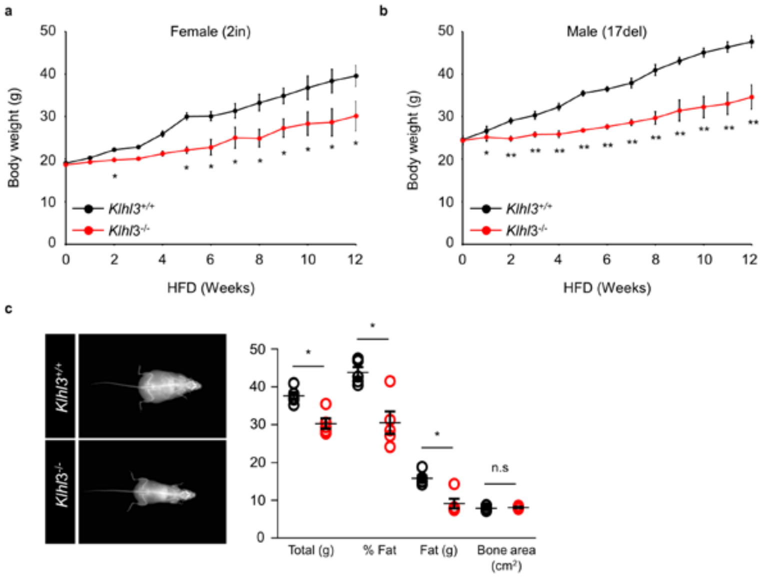

Supplementary Fig. 3

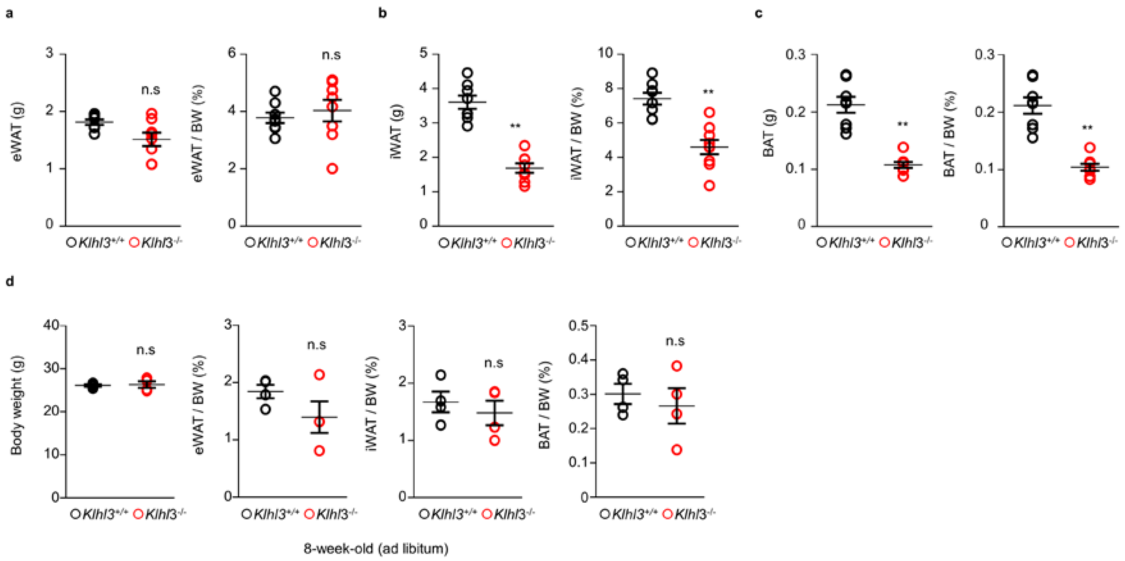

Supplementary Fig. 4

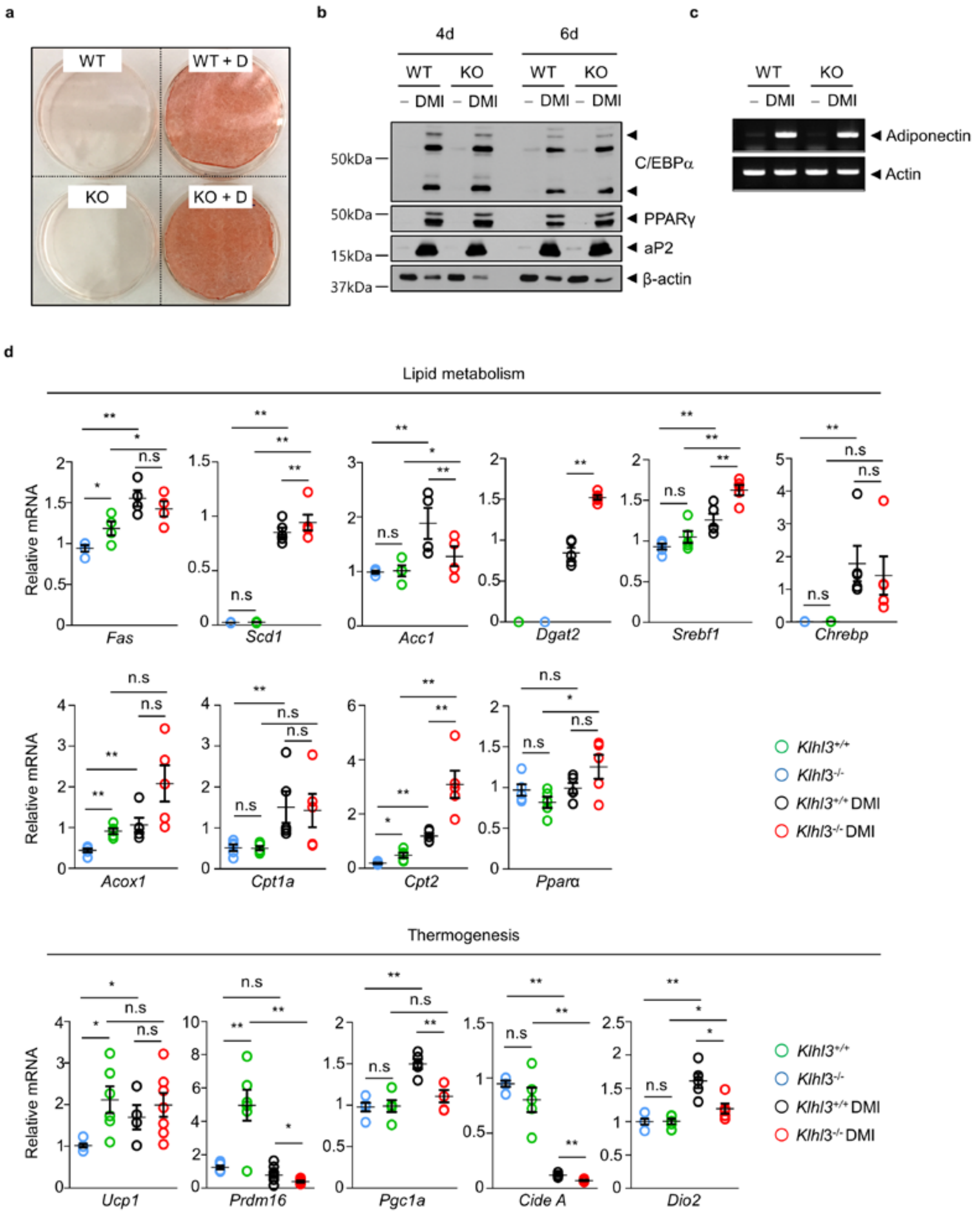

Supplementary Fig. 5

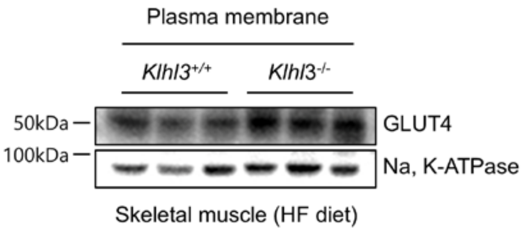

Supplementary Fig. 6

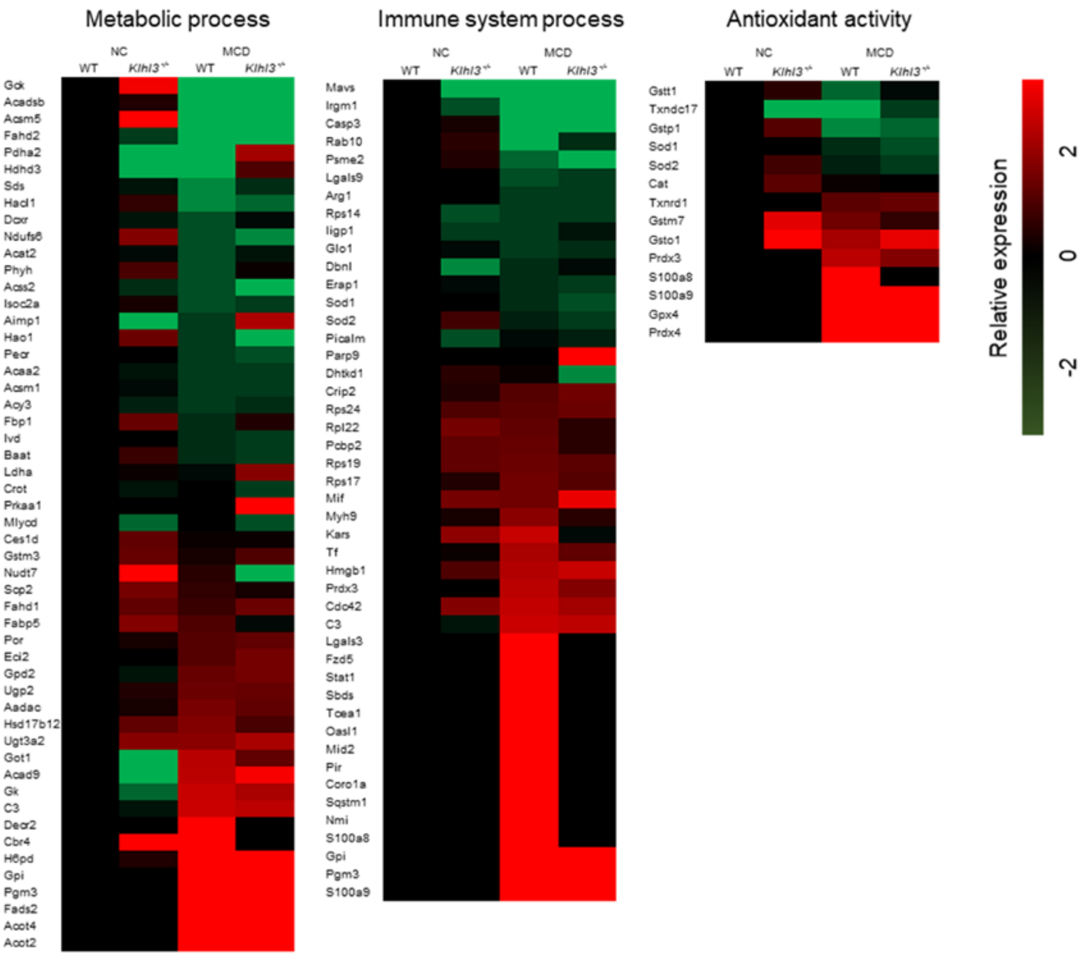

Supplementary Fig. 7

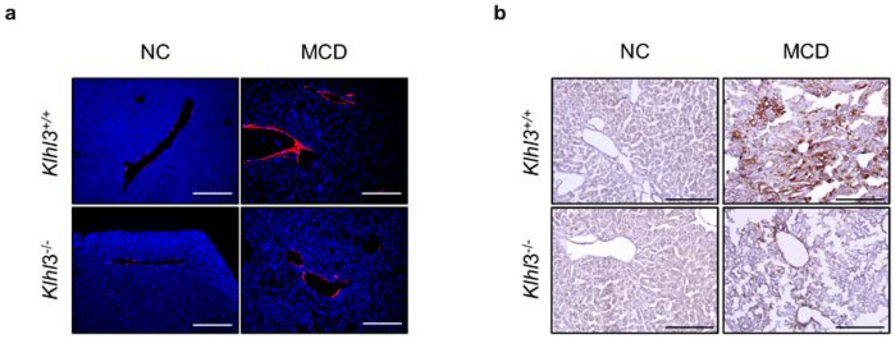

Supplementary Fig. 8

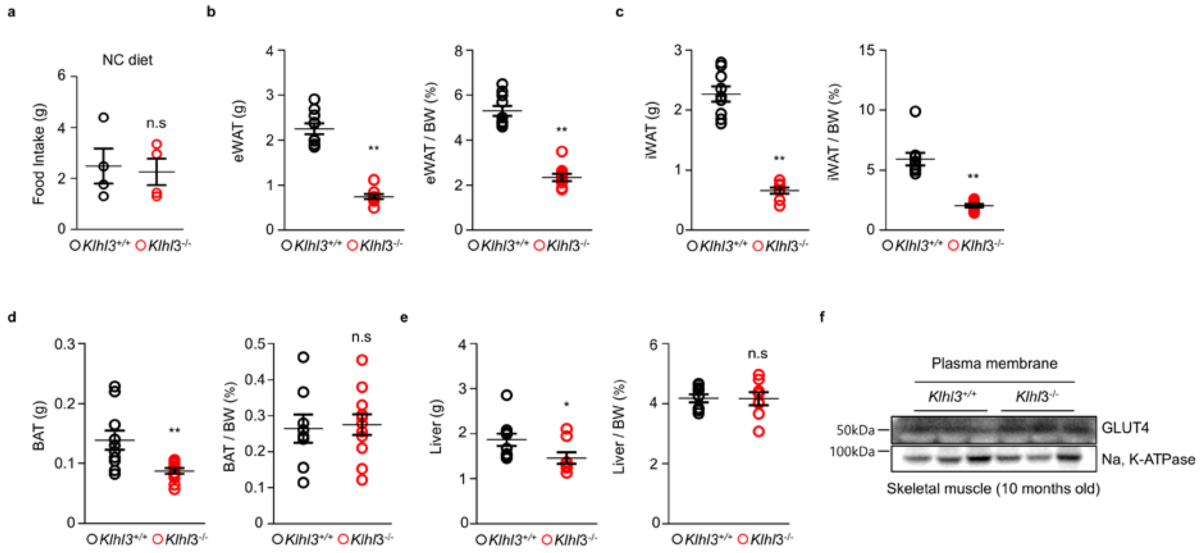

Supplementary Fig. 9

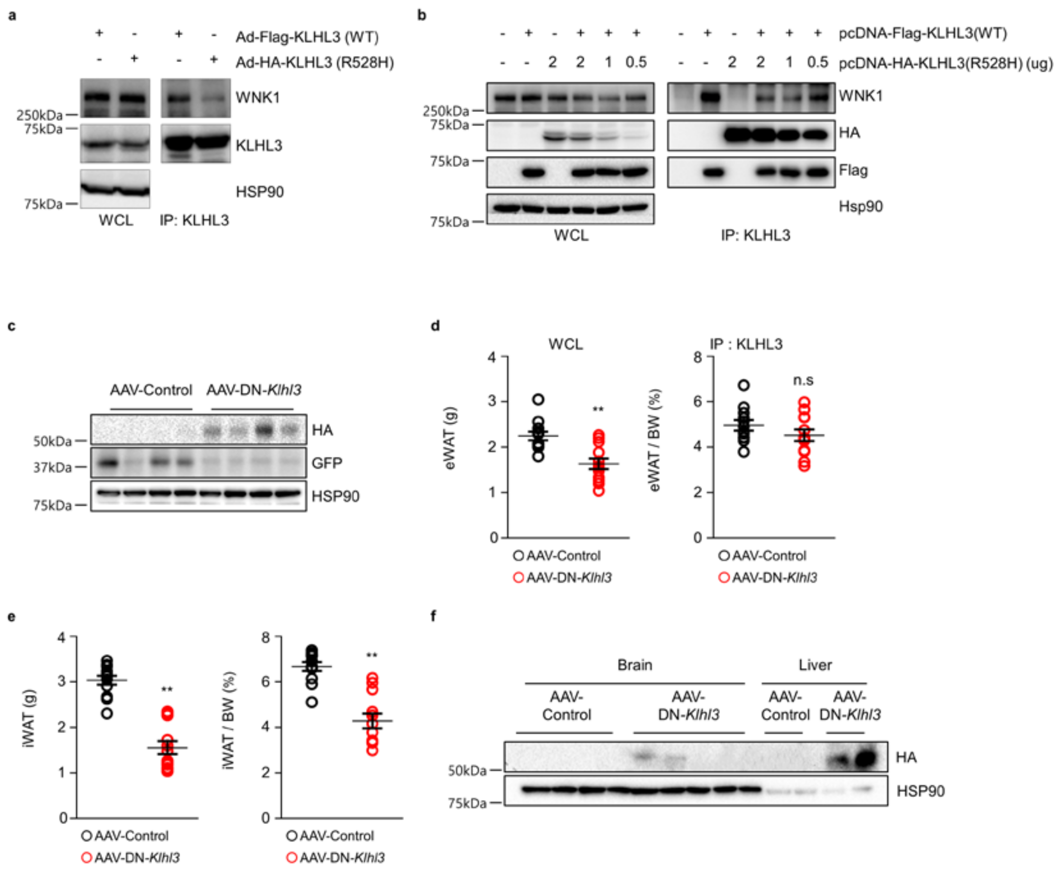

Supplementary Fig. 10

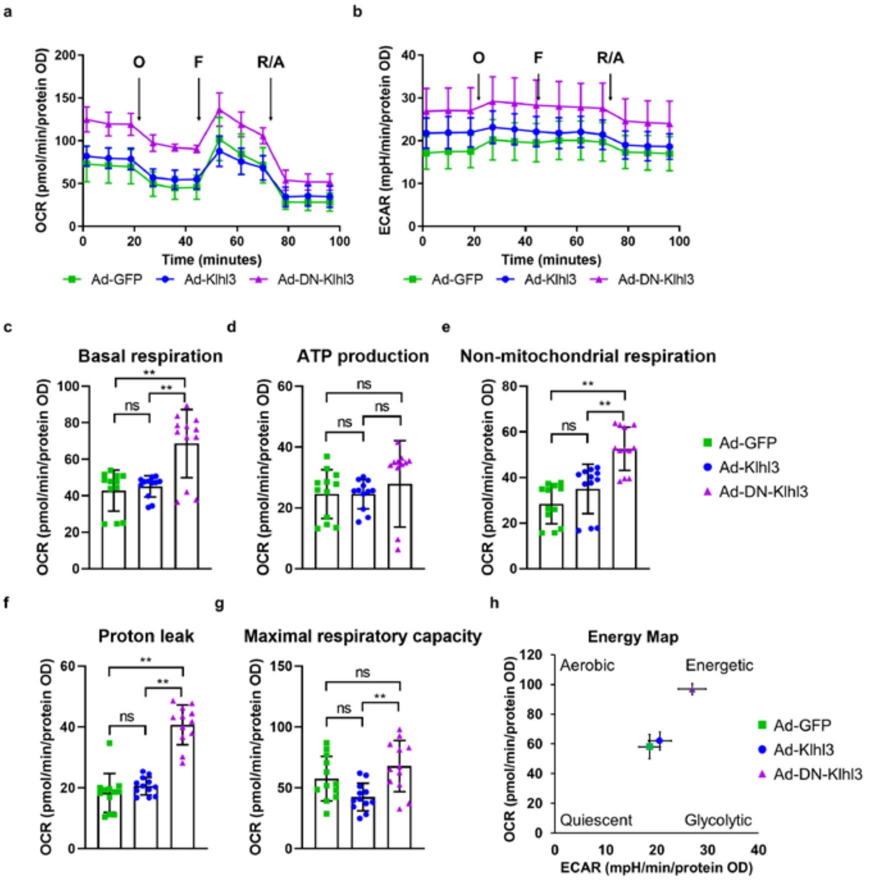

Supplementary Fig. 11

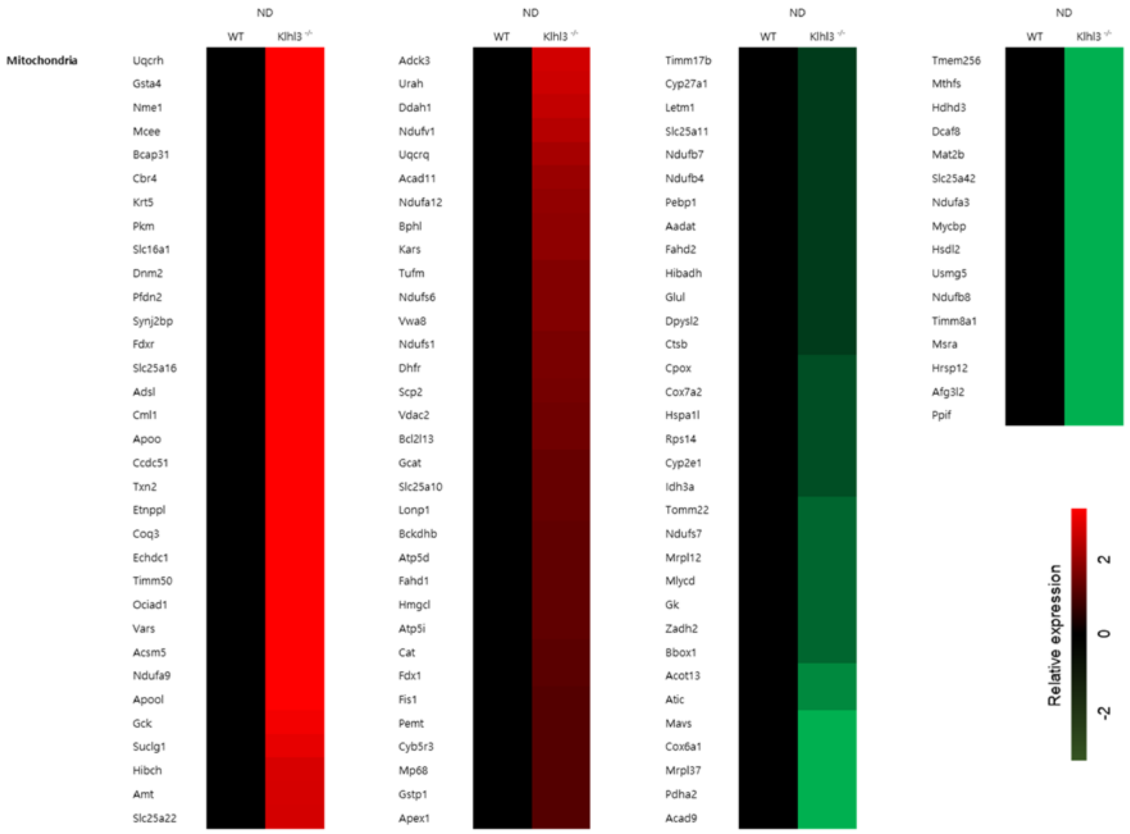

Supplementary Fig. 12

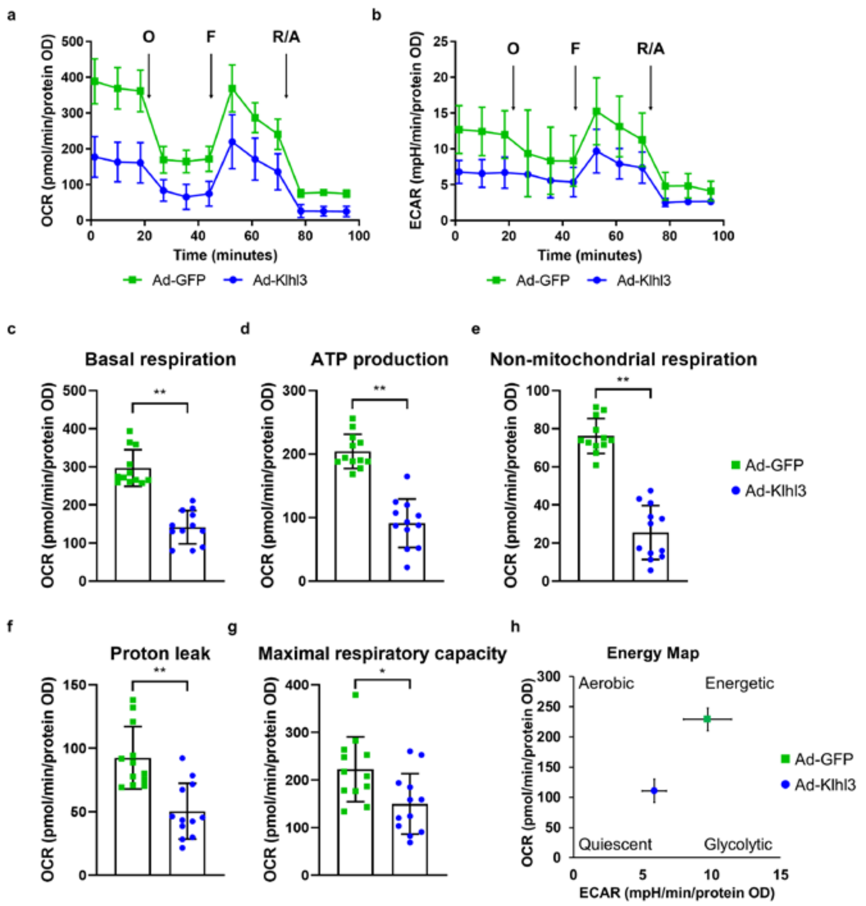

Supplementary Fig. 13

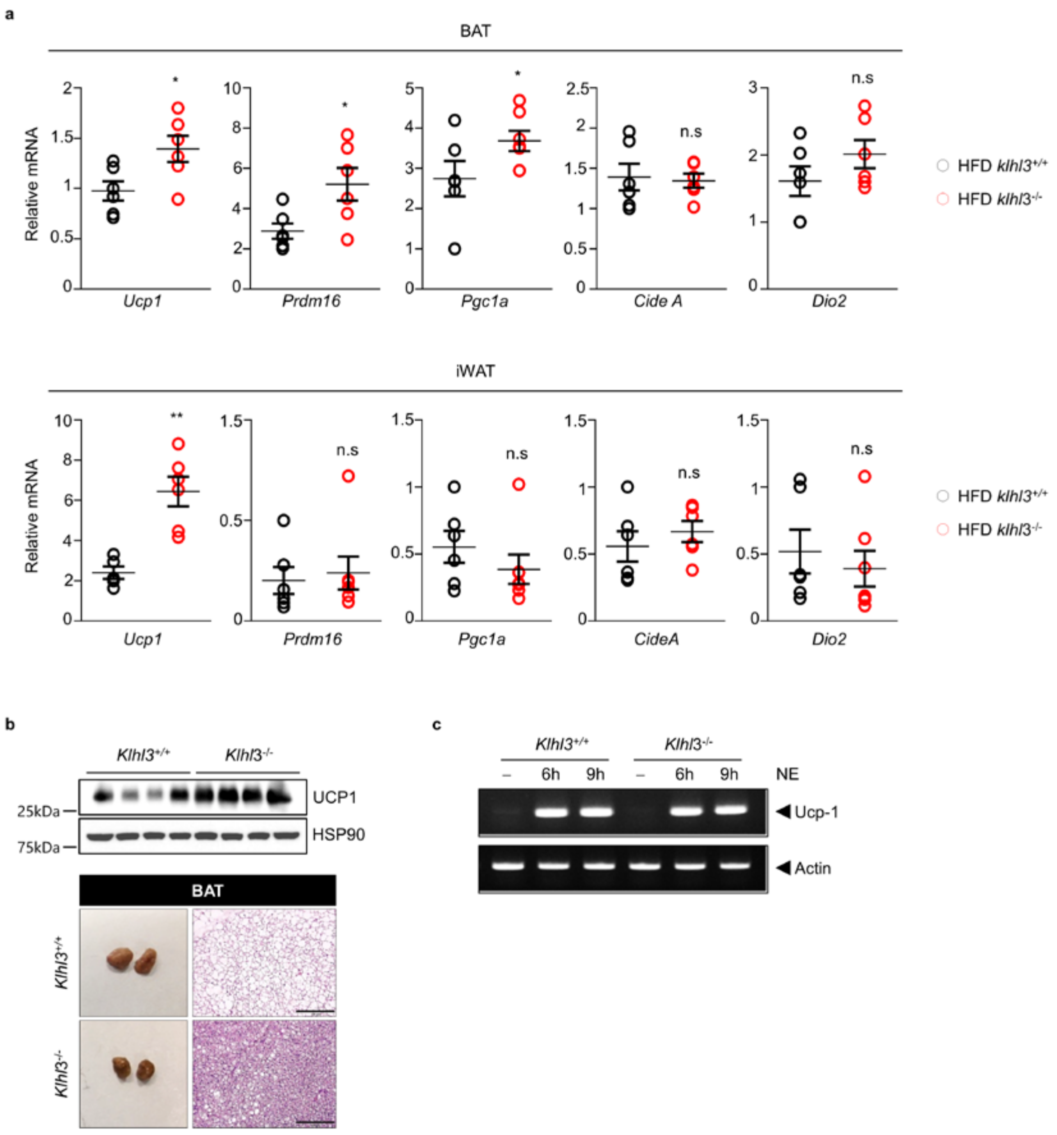

Supplement: Supplementary file 1 — Supplementary data [file 12276_2022_833_MOESM1_ESM.pdf]
